# A Deep Learning Framework for the Discovery of Natural-Product Candidate Binders of Acetyl-CoA Carboxylase 2 (ACC2) with Potential Relevance to Cardiometabolic Lipid Metabolism

**DOI:** 10.3390/ph19071123

**Published:** 2026-07-21

**Authors:** Nada A. Alzunaidy

**Affiliations:** Department of Food Science and Human Nutrition, College of Agriculture and Food, Qassim University, Buraydah 51452, Saudi Arabia; n.alznedy@qu.edu.sa

**Keywords:** antioxidant, obesity, insulin resistance, oxidative stress, acetyl-CoA carboxylase 2, lipid metabolism, dietary bioactive compounds, deep learning, graph neural network, molecular docking, molecular dynamics

## Abstract

**Background/Objectives:** Obesity and related metabolic diseases arise from an interplay of lipid overload, insulin resistance and oxidative stress. Acetyl-CoA carboxylase 2 (ACC2) controls malonyl-CoA production and thereby gates mitochondrial fatty-acid oxidation, placing it at the intersection of lipid handling and redox-sensitive metabolic dysfunction. Dietary antioxidants such as polyphenols, flavonoids and terpenoids are increasingly studied as modulators of these pathways, yet systematic prioritization of food-derived antioxidant compounds against defined metabolic targets remains challenging. We developed an integrated deep learning and structure-based workflow to prioritize FooDB compounds with predicted ACC2-binding potential. **Methods:** A curated set of 3983 ACC2 bioactivity records from ChEMBL 36 was used to train scaffold-split models, including graph neural-network and graph–Morgan fingerprint-fusion architectures. The calibrated ensemble screened 139,988 FooDB compounds; 200 candidates with predicted activity probability above 0.70 were docked against the ACC2 carboxyltransferase domain (PDB ID: 3FF6), and six prioritized complexes underwent 500 ns molecular dynamics and MM/GBSA analysis. **Results:** Redocking of the co-crystallized ligand reproduced the experimental pose (RMSD 1.2 Å). Although the highest-ranked screening hits were antioxidant terpenoids and alkaloids, docking-based prioritization from the top candidates selected six larger, more polar food-derived compounds, including glycosides and two nucleotide/cofactor-like conjugates, which showed docking scores from −7.47 to −6.65 kcal/mol versus −6.21 kcal/mol for the reference ligand. Glu539 emerged as a recurrent interaction hotspot. All candidates gave more favourable MM/GBSA binding free energies than the reference (ΔG = −22.52 kcal/mol), led by FDB029596 (−35.65), FDB021568 (−34.14) and FDB017807 (−33.87 kcal/mol). **Conclusions:** This workflow provides a reproducible framework for prioritizing food-derived compounds as candidate ACC2 binders relevant to obesity and metabolic disease, generating structurally supported hypotheses for biochemical and nutritional validation. The prioritized compounds are computational candidates only and require biochemical and cellular (experimental) validation before any ACC2-related biological relevance can be established.

## 1. Introduction

Obesity, type 2 diabetes and cardiovascular disease continue to rise worldwide and are commonly attributed to excess energy intake, macronutrient imbalance and reduced physical activity [1]. Beyond energy balance, however, the way absorbed nutrients are stored, oxidized or redirected between metabolic pathways is governed by defined molecular control points [2]. Lipid metabolism is particularly sensitive to nutritional state, because fatty acids act both as energy substrates and as storage molecules depending on hormonal, cellular and enzymatic context [3]. After feeding, fatty-acid synthesis, mitochondrial fatty-acid oxidation and lipid storage are dynamically adjusted to nutrient availability [4]. When this balance is disrupted, ectopic lipid accumulation, impaired substrate flexibility and altered energy homeostasis develop; these changes are frequently accompanied by oxidative stress, an imbalance between reactive oxygen species production and antioxidant defences that promotes adipose tissue dysfunction, insulin resistance and chronic low-grade inflammation [5]. Connecting food-derived chemical diversity with the enzymes and pathways that regulate metabolic flux is therefore central to nutritional biochemistry [6]. Foods supply not only macronutrients but also diverse small molecules, including phenolics, flavonoids, fatty-acid derivatives, alkaloids, terpenoids and other bioactive constituents, many of which possess well-documented antioxidant activity [7]. These compounds may influence metabolic regulation through direct or indirect interactions with proteins involved in nutrient sensing, lipid handling and energy utilization [8]. Food-composition data alone cannot define these molecular contributions, because structural diversity, concentration, bioavailability and enzyme-level interaction potential vary substantially across food-derived molecules [9]. Computational prioritization provides a framework for organizing this chemical complexity and identifying compounds that warrant nutritional and biochemical investigation [10]. This approach is especially relevant for enzymes positioned at the interface between nutrient availability, lipid oxidation and redox balance, where changes in enzymatic regulation may influence cellular responses to dietary substrates [11].

Acetyl-CoA carboxylase 2 (ACC2) is a nutrient-responsive enzymatic checkpoint in lipid metabolism that links cellular nutrient status to mitochondrial fatty-acid oxidation [12]. ACC2 catalyses the carboxylation of acetyl-CoA to malonyl-CoA at the mitochondrial surface, where malonyl-CoA regulates carnitine palmitoyltransferase-1, the rate-limiting gateway for long-chain fatty-acid entry into β-oxidation [13]. Through this axis, ACC2 contributes to the partitioning of fatty acids between mitochondrial oxidation and lipid storage [14]. In nutrient-replete states, increased malonyl-CoA availability can restrict fatty-acid transport into mitochondria, whereas reduced malonyl-CoA availability favours fatty-acid oxidation [15]. Because chronically elevated malonyl-CoA and suppressed fatty-acid oxidation contribute to lipid accumulation, insulin resistance and the oxidative burden associated with obesity, ACC2 has attracted sustained interest as a metabolically relevant enzyme, particularly in tissues with high oxidative demand such as skeletal muscle, heart and liver [16]. From a nutritional perspective, ACC2 is relevant not primarily as a pharmacological target but as an enzyme that helps determine how dietary and stored fatty acids are allocated between oxidation and accumulation [14]. Dietary patterns, fasting–feeding transitions and food-derived metabolites can influence lipid metabolism through networks involving acetyl-CoA availability, malonyl-CoA signalling, mitochondrial oxidation and cellular energy sensing [17]. Assessing whether food-derived compounds contain structural features compatible with ACC2 interaction may therefore generate hypotheses about nutritional modulation of lipid-handling pathways [18]. These hypotheses require cautious interpretation: computationally predicted interaction potential does not establish physiological efficacy, bioavailability or in vivo modulation [19]. ACC2 nonetheless provides a mechanistically coherent target for computational nutritional biochemistry, because its activity is closely connected to substrate switching and fatty-acid utilization [13].

Dietary compounds can participate in metabolic regulation beyond their contribution to energy intake [20]. Food matrices contain diverse small molecules, including phenolics, flavonoids, lipids, organic acids, terpenoids and other secondary metabolites, many of which are recognized dietary antioxidants and occupy chemical space compatible with protein interaction [21]. These compounds may influence nutrient-responsive pathways through enzyme binding, redox activity, membrane effects or signalling-associated mechanisms [22]. Because oxidative stress and impaired mitochondrial fatty-acid oxidation are mechanistically coupled in obesity and insulin resistance, redox-active dietary compounds that may also engage lipid-handling enzymes such as ACC2 are of particular interest, providing the rationale for prioritizing antioxidant phytochemicals in this ACC2-directed screen [5]. In lipid metabolism, this chemical diversity is relevant because fatty-acid utilization is controlled by coordinated enzymatic checkpoints that respond to nutritional and redox state. ACC2 is positioned within this regulatory network by controlling malonyl-CoA availability and mitochondrial fatty-acid entry, making it a suitable enzyme for examining diet-associated molecular interactions relevant to obesity and metabolic disease [13]. However, the breadth of food-derived chemical space remains difficult to investigate experimentally at scale. Systematic prioritization is therefore needed to identify antioxidant and other dietary compounds with structural properties consistent with ACC2 interaction before targeted biochemical or nutritional evaluation [23]. A nutrition-oriented computational framework can connect curated enzyme-activity data with food-compound chemical structures and generate a ranked set of dietary molecules for downstream structural and mechanistic assessment [24].

Here, we developed an integrated computational nutritional biochemistry workflow to prioritize food-derived antioxidant compounds with predicted ACC2 interaction potential. ACC2 bioactivity records from ChEMBL 36 [25] were curated, standardized into pIC50-based regression and activity-classification datasets, and used to train scaffold-split machine-learning models based on molecular fingerprints, molecular graphs and graph–Morgan late fusion. The trained models were applied to FooDB [26] to rank dietary compounds by predicted ACC2 activity propensity and ensemble uncertainty. Top-ranked FooDB candidates were further evaluated by molecular docking against the ACC2 carboxyltransferase-domain structure 3FF6, followed by molecular dynamics simulations and post-simulation analyses of complex stability, residue-level interactions and binding free energy. This workflow provides a computational framework for prioritizing candidate dietary bioactives, many of them established antioxidants, with predicted relevance to ACC2-associated lipid metabolic regulation, and it generates structurally supported hypotheses for future biochemical and nutritional validation in the context of obesity and metabolic disease.

## 2. Results

### 2.1. Curated ACC2 Data Reveal Structured Class Imbalance

**Figure 1:**
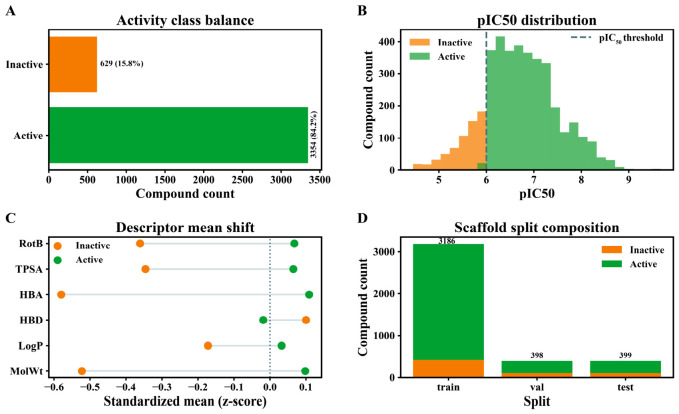
Curated ACC2 bioactivity dataset and scaffold-based data partitioning. (**A**) Activity-class distribution of the curated ACC2 dataset, showing 629 inactive compounds and 3354 active compounds. (**B**) pIC50 distribution of active and inactive compounds, with the activity threshold indicated at pIC50 6.0. (**C**) Standardized mean differences in selected molecular descriptors between active and inactive compounds. (**D**) Composition of the scaffold-based train, validation and test split, containing 3186, 398 and 399 compounds, respectively.

To construct an ACC2-focused modelling dataset, bioactivity records were curated from ChEMBL 36 and restricted to compounds with valid canonical SMILES, quantitative IC50 values and scaffold-split assignments. After standardization and duplicate handling, the final dataset contained 3983 compounds. Activity classification using a pIC50 threshold of 6.0 assigned 3354 compounds as active and 629 compounds as inactive, corresponding to 84.2% and 15.8% of the dataset, respectively (Figure 1A). This active-enriched ratio reflects the reporting bias of public ACC2 bioactivity data toward confirmed active compounds and was addressed during modelling through balanced class weighting, validation-locked threshold selection and probability calibration rather than by resampling. The pIC50 distribution separated around the activity threshold, with inactive compounds enriched below pIC50 6.0 and active compounds distributed predominantly above this cutoff (Figure 1B). Descriptor-level comparison between active and inactive compounds showed systematic differences in molecular properties across the two classes. Standardized mean shifts were observed for molecular weight, LogP, hydrogen-bond donors, hydrogen-bond acceptors, topological polar surface area and rotatable bonds, indicating that activity labels were associated with measurable physicochemical differences rather than only the pIC50 threshold definition (Figure 1C). These patterns supported the use of both fingerprint-based and graph-based molecular representations for downstream modelling. A scaffold-based split was used to reduce structural leakage between model-development and test partitions. The representative split contained 3186 training compounds, 398 validation compounds and 399 test compounds (Figure 1D). Across the three scaffold-split seeds, split sizes were unchanged, with 3186 training compounds, 398 validation compounds and 399 test compounds in each seed (Tables S1 and S2). The training set contained 494 unique scaffolds, whereas the validation and test sets contained 398 and 399 unique scaffolds, respectively (Tables S1 and S2). No scaffold overlap was detected between the training and test sets across the three seeds, with a train–test scaffold-overlap count of 0 and an overlap fraction of 0.0 (Tables S1 and S2). The scaffold distribution contained 1291 total scaffolds, with a mean scaffold size of 3.08 compounds, a median scaffold size of 1.0, a maximum scaffold size of 171.0 and a 95th percentile scaffold size of 9.0 (Table S1). The largest scaffold contained 171 compounds, followed by scaffolds containing 151, 87, 81 and 66 compounds (Table S3). Split-size diagnostics confirmed that training, validation and test sizes remained stable across seeds (Figure S1A). Graph-size distributions based on atom and directed-edge counts were broadly comparable across the training, validation and test partitions for each seed, supporting consistent molecular graph construction across scaffold-split subsets (Figure S1B–G; Tables S4–S6).

### 2.2. Fingerprint and Graph Models Benchmark ACC2 pIC50 Prediction Across Scaffold Splits

**Figure 2:**
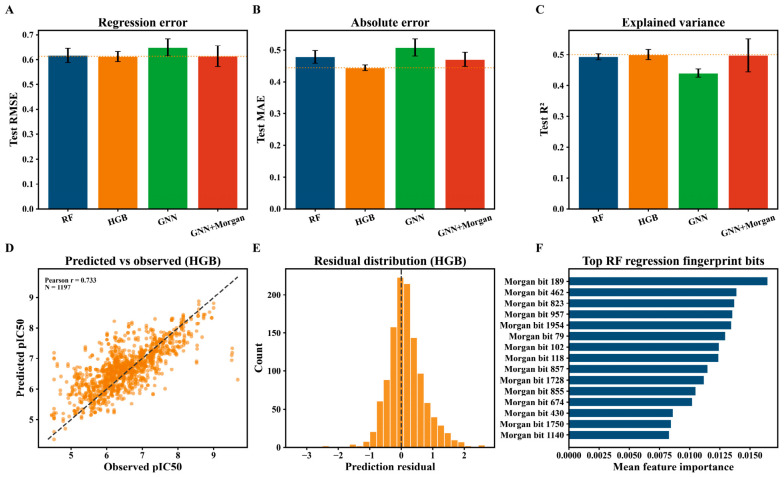
Scaffold-split regression performance for ACC2 pIC50 prediction. (**A**–**C**) Test-set root-mean-square error, mean absolute error and coefficient of determination for random forest, histogram gradient boosting, graph neural network and graph–Morgan fusion models across independent scaffold-split seeds. Bars show mean performance, and error bars indicate variability across seeds. Dashed horizontal lines indicate the reference performance level used for visual comparison. (**D**) Predicted versus observed pIC50 values for the histogram gradient boosting model on the combined test predictions across seeds. (**E**) Distribution of prediction residuals for the histogram gradient boosting model. (**F**) Mean feature importance of the top-ranked Morgan fingerprint bits from the random forest regression model.

To evaluate continuous ACC2 activity prediction, pIC50 regression models were trained and tested under the fixed scaffold-split scheme. Four model classes were compared: random forest using Morgan fingerprints, histogram gradient boosting using Morgan fingerprints, a graph neural network, and a graph–Morgan late-fusion model. Test-set performance was assessed across independent scaffold-split seeds using root-mean-square error, mean absolute error and coefficient of determination, with full per-seed metrics reported in Table S7 and paired test-set comparisons reported in Table S8. Across the evaluated models, test-set root-mean-square error values clustered within a similar range, indicating broadly comparable scaffold-split regression performance (Figure 2A). Histogram gradient boosting showed the lowest mean absolute error among the four models, whereas the graph neural-network model showed higher mean absolute error and lower explained variance than the fingerprint-based baselines (Figure 2B,C). The graph–Morgan late-fusion model produced regression errors close to the fingerprint baselines and showed explained variance comparable to the random forest and histogram gradient boosting models, although coefficient-of-determination values varied more across seeds (Figure 2A–C). These results indicate that graph-based molecular representations did not consistently outperform Morgan fingerprint baselines for ACC2 pIC50 prediction under scaffold splitting. Because histogram gradient boosting gave the most favourable overall regression profile among the tested models, its predictions were further inspected against observed pIC50 values. The aggregated test-set predicted-versus-observed plot showed a positive relationship between predicted and measured pIC50 values, with Pearson r = 0.73 across *n* = 1197 test predictions (Figure 2D). The corresponding residual distribution was centred close to zero, indicating limited systematic bias in the overall prediction direction, although residual spread remained evident across scaffold-held-out test compounds (Figure 2E). Random forest feature-importance analysis identified a ranked set of Morgan fingerprint bits contributing to the regression model, with Morgan bit 189 showing the highest mean feature importance among the displayed descriptors (Figure 2F). Per-seed diagnostic plots supported the aggregate trends. Random forest predictions showed consistent positive associations between observed and predicted pIC50 values across the three scaffold-split seeds, with residual distributions centred near zero but with outlying errors in each split (Figure S2). Histogram gradient boosting showed similar per-seed behaviour, with predicted-versus-observed plots following the diagonal trend and residual histograms concentrated around zero across all three seeds (Figure S3). The graph neural-network model showed convergence of training loss across seeds, but validation loss remained higher than training loss, consistent with the more limited test-set performance observed in the aggregate regression metrics (Figure S4). The graph–Morgan fusion model also converged across seeds and produced test-set prediction trends similar to the graph-only model, with residual distributions centred around zero but retaining outliers at both ends of the residual range (Figure S5). Together, these analyses show that ACC2 pIC50 prediction was feasible under scaffold splitting, but model performance was constrained by chemical-structure generalization. Fingerprint-based models, particularly histogram gradient boosting, provided the strongest regression benchmark in this comparison, whereas graph and graph–Morgan models provided complementary molecular representations for downstream classification, calibration and FooDB screening analyses.

### 2.3. Validation-Locked Classification Supports ACC2 Activity Discrimination Across Scaffold Splits

**Figure 3:**
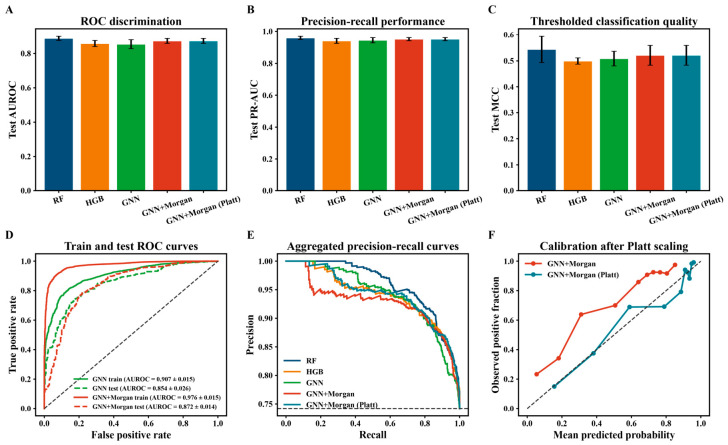
Scaffold-split classification and probability calibration for ACC2 activity prediction. (**A**–**C**) Test-set area under the receiver operating characteristic curve, precision–recall area under the curve and Matthews correlation coefficient for random forest, histogram gradient boosting, graph neural-network, graph–Morgan fusion and Platt-calibrated graph–Morgan fusion classifiers. Bars show mean performance across scaffold-split seeds, and error bars indicate variability across seeds. (**D**) Aggregated train and test receiver operating characteristic curves for the graph neural-network and graph–Morgan fusion classifiers. (**E**) Aggregated precision–recall curves for the evaluated classifiers. (**F**) Reliability curve comparing graph–Morgan fusion probabilities before and after Platt scaling.

Binary ACC2 activity classification was evaluated using the same scaffold-split framework applied to pIC50 regression. Models were trained to distinguish active from inactive compounds using validation-selected probability thresholds, and test-set performance was assessed using threshold-independent metrics, thresholded classification metrics and calibration diagnostics. Full per-seed performance values are reported in Table S7, and paired test-set comparisons are reported in Table S8. All evaluated classifiers showed above-chance discrimination on scaffold-held-out test sets (Figure 3A). Random forest produced the highest mean test area under the receiver operating characteristic curve among the displayed models. The graph–Morgan fusion classifier showed test AUROC values of 0.88, 0.85 and 0.87 across seeds 1–3, corresponding to a mean test AUROC of 0.87 ± 0.01 (Figure 3A,D and Figure S9; Table S7). The graph-only model showed a mean test AUROC of 0.85 ± 0.02, indicating that late fusion with Morgan fingerprints improved test-set discrimination relative to the graph-only classifier in this comparison (Table S7). However, paired testing across the three scaffold seeds did not show a statistically robust difference after false-discovery-rate correction for AUROC (paired *t*-test q = 0.27; Wilcoxon q = 0.34), indicating that this improvement should be interpreted as a trend rather than a confirmed model-level advantage (Table S8). Precision–recall analysis was used because the curated ACC2 dataset was class-imbalanced, with active compounds forming the majority class. All models retained high test precision–recall performance (Figure 3B,E). The graph–Morgan fusion model achieved test precision–recall area under the curve values of 0.95, 0.93 and 0.95 across the three seeds, corresponding to a mean of 0.95 ± 0.01. The graph-only model achieved a mean test precision–recall area under the curve of 0.94 ± 0.01 (Table S7). As with AUROC, the paired comparison did not remain significant after correction (paired *t*-test q = 0.28; Wilcoxon q = 0.34), supporting comparable precision–recall performance between the graph-only and fusion classifiers under scaffold splitting (Table S8). Thresholded classification metrics showed moderate activity-class assignment performance after validation-locked threshold selection (Figure 3C). The graph–Morgan fusion classifier produced test Matthews correlation coefficient values of 0.54, 0.47 and 0.53 across seeds, giving a mean of 0.52 ± 0.03 (Table S7). The corresponding mean balanced accuracy was 0.75 ± 0.02, and the mean F1 score was 0.88 ± 0.01 (Table S7). Compared with the graph-only classifier, the fusion model showed a higher mean F1 score but lower mean balanced accuracy, reflecting a shift in the trade-off between active-compound recovery and inactive-compound discrimination (Table S7). This trade-off was also reflected in the confusion-matrix counts: the fusion model produced fewer false negatives on average than the graph-only model, but more false positives (Table S7). Per-seed diagnostics supported the aggregate classification trends. Random forest showed test AUROC values of 0.89, 0.87 and 0.89, with precision–recall average precision values of 0.96, 0.94 and 0.96 across seeds 1–3 (Figure S6). Histogram gradient boosting showed test AUROC values of 0.87, 0.84 and 0.85, with precision–recall average precision values of 0.95, 0.92 and 0.94 (Figure S7). The graph neural-network classifier showed test AUROC values of 0.87, 0.82 and 0.85 and precision–recall average precision values of 0.95, 0.92 and 0.95 (Figure S8). The graph–Morgan fusion classifier showed test AUROC values of 0.88, 0.85 and 0.87 across the three seeds, while its training AUROC values were higher at 0.95, 0.98 and 0.98, indicating stronger fit to the training distribution than to scaffold-held-out compounds (Figure S9). Because predicted probabilities were later used for FooDB ranking and uncertainty filtering, calibration was assessed for the graph–Morgan fusion classifier. Platt scaling improved the alignment between predicted probabilities and observed positive fractions in the validation-set reliability diagrams, while leaving the test receiver operating characteristic curves unchanged, as expected for a monotonic calibration transform (Figure 3F and Figure S10). In the Platt-scaled test diagnostics, AUROC values remained 0.88, 0.85 and 0.87 across seeds 1–3 (Figure S10). Probability histograms showed that calibration shifted the distribution of predicted probabilities, particularly for high-confidence active predictions, supporting its use before downstream probability-based screening (Figure S10). Together, these results show that ACC2 activity classification was feasible under scaffold-held-out evaluation, with all model classes achieving above-chance discrimination and high precision–recall performance. The graph–Morgan fusion classifier provided calibrated probability outputs and reduced false negatives relative to the graph-only model, making it suitable for ensemble-based FooDB screening. However, paired statistical comparisons across the three seeds did not support a corrected significant difference between graph-only and graph–Morgan fusion performance. Subsequent screening results should therefore be interpreted as computational prioritization rather than experimentally validated ACC2 modulation.

### 2.4. Applicability-Domain Analysis Prioritizes FooDB Compounds for ACC2-Focused Validation

**Figure 4:**
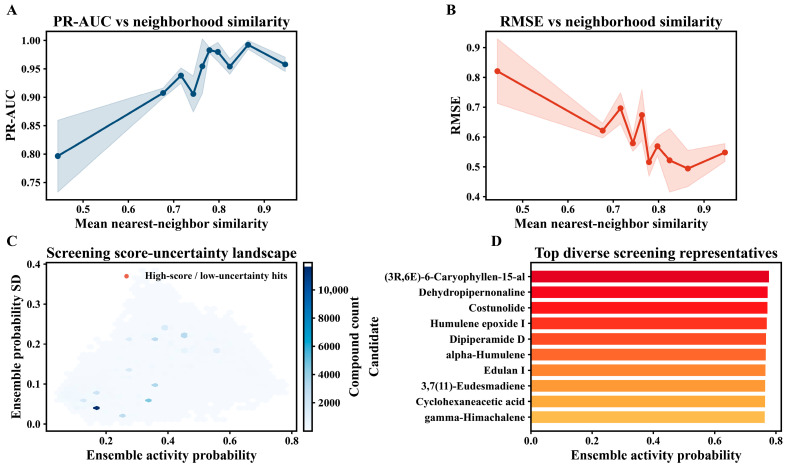
Applicability-domain-guided FooDB screening for predicted ACC2 activity. (**A**) Precision–recall area under the curve as a function of mean nearest-neighbour similarity to the ACC2 training set. Points indicate similarity-bin performance, and shaded regions indicate variability across scaffold-split seeds. (**B**) Root-mean-square error as a function of mean nearest-neighbour similarity to the training set. (**C**) Ensemble screening score–uncertainty landscape for FooDB compounds, showing ensemble activity probability against ensemble probability standard deviation. The highlighted region indicates high-score, low-uncertainty candidates. (**D**) Top scaffold-diverse FooDB screening representatives ranked by ensemble activity probability.

To assess whether model performance depended on chemical similarity to the training set, the graph–Morgan fusion model was evaluated within an applicability-domain framework based on maximum Morgan Tanimoto similarity to training compounds. Using an applicability-domain threshold of 0.35, 397, 396 and 395 test compounds were retained inside the domain for seeds 1–3, respectively, from 399 test compounds per seed (Table S9). Within this domain, the model achieved mean classification performance values of AUROC = 0.87, precision–recall area under the curve = 0.95, Matthews correlation coefficient = 0.52, balanced accuracy = 0.75 and F1 score = 0.88 across the three seeds (Table S9). Regression performance within the same applicability domain gave mean root-mean-square error = 0.60, mean absolute error = 0.46 and coefficient of determination = 0.50 (Table S9). Chemical-similarity diagnostics showed that most scaffold-held-out test compounds retained moderate to high nearest-neighbour similarity to the training set, with the main density concentrated around maximum Tanimoto similarity values of approximately 0.7–0.85 across seeds (Figure S11A–C). Two-dimensional chemical-space projections showed that test compounds occupied continuous regions rather than isolated clusters, with local variation in nearest-neighbour similarity across the projected space (Figure S11D–F; Table S11). Prediction-space projections further showed that both classification probabilities and predicted pIC50 values varied across the same latent chemical-space structure, indicating that model outputs were not uniformly distributed across the test chemical space (Figure S12; Table S11). Performance stratification by chemical-similarity bins indicated that prediction quality was generally higher for compounds with greater neighbourhood similarity to the training set. Precision–recall area under the curve increased from a mean value of 0.79 in the lowest similarity bin to values above 0.95 in several higher-similarity bins (Figure 4A and Figure S13A–C; Table S10). Regression error showed the opposite trend, with mean root-mean-square error decreasing from 0.82 in the lowest similarity bin to lower values in higher-similarity regions, including 0.49 in the bin centred at 0.86 (Figure 4B and Figure S13D–F; Table S10). These results indicate that classification and regression predictions were more reliable for FooDB-like candidates located closer to the ACC2 training chemical space. The calibrated graph–Morgan fusion ensemble was then applied to FooDB compounds to prioritize dietary molecules with predicted ACC2 activity propensity. Ensemble screening ranked compounds by mean predicted activity probability, with ensemble standard deviation used as an uncertainty estimate. The score–uncertainty landscape identified a high-score, low-uncertainty region used to define candidates for downstream structural assessment (Figure 4C). The top-ranked FooDB compounds included (3R,6E)-6-caryophyllen-15-al, dehydropipernonaline, costunolide, humulene epoxide I, humulene epoxide, α-humulene epoxide, dipiperamide D, α-humulene, edulan I and 3,7(11)-eudesmadiene, with ensemble activity probabilities ranging from 0.77 to 0.78 among the top ten ranked compounds (Figure 4D; Table S12). Notably, several of these top-ranked candidates, including the sesquiterpenes caryophyllene, humulene and costunolide and the piperamide alkaloids, are recognized dietary antioxidants, consistent with the special relevance of redox-active food compounds to lipid-metabolic regulation. Here, antioxidant status is assigned from published reports for these chemical classes rather than from antioxidant activity measured in this study, and it was not itself a criterion for selecting the six compounds advanced to docking. The same top-ranked compounds were retained in the high-confidence uncertainty-filtered subset, with ensemble standard deviations ranging from 0.08 to 0.10 across the top ten candidates (Table S13). Scaffold-diverse representative selection reduced redundancy among highly ranked FooDB compounds while retaining candidates with high predicted activity probabilities. The top scaffold-diverse representatives included (3R,6E)-6-caryophyllen-15-al, dehydropipernonaline, costunolide, humulene epoxide I, dipiperamide D, α-humulene, edulan I, 3,7(11)-eudesmadiene, cyclohexaneacetic acid and γ-himachalene, with ensemble activity probabilities ranging from 0.76 to 0.78 (Figure 4D; Table S14). Physicochemical profiling showed that the top-ranked candidates covered a range of molecular sizes and polarity values, including (3R,6E)-6-caryophyllen-15-al with molecular weight 220.356, LogP 3.98 and topological polar surface area 17.07, and dipiperamide D with molecular weight 596.72, LogP 5.93 and topological polar surface area 77.54 (Table S15). These property differences indicate that the prioritized FooDB set contains chemically diverse candidates rather than a single physicochemical class. Classification and regression error analyses identified compounds for which model predictions were less reliable. Hard false-positive compounds, hard false-negative compounds and compounds with the largest pIC50 regression errors are listed in Tables S16–S18. These error sets provide a reference for interpreting downstream screening results and for identifying molecular regions where the model may overestimate or underestimate ACC2 activity. Atom-level attribution was calculated for representative interpreted compounds to visualize substructures contributing to classification outputs (Figure S14; Table S19). These attribution maps provide model-interpretability support but should be interpreted as computational explanations of model behaviour rather than mechanistic evidence of ACC2 binding or modulation.

### 2.5. FooDB Screening and Docking Prioritize Candidate ACC2 Binders

**Figure 5:**
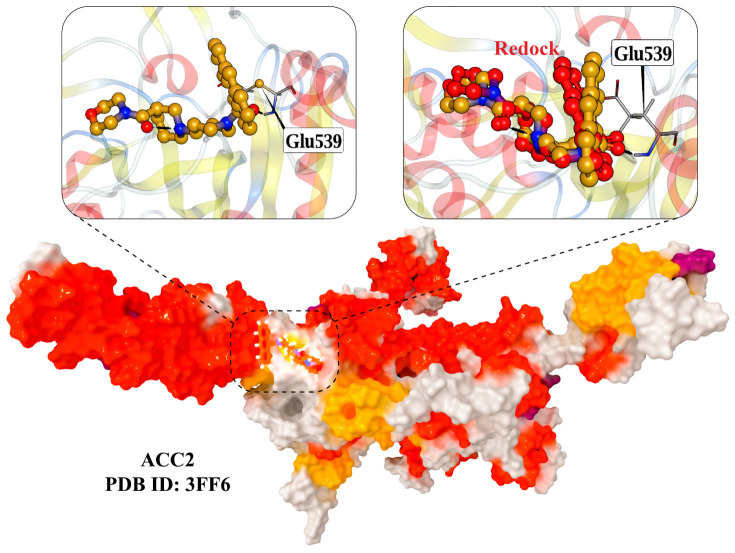
Surface representation of ACC2 (PDB ID: 3FF6) highlighting the ligand-binding pocket used for docking. The dashed box indicates the binding region corresponding to the co-crystallized ligand site. Upper panels show the binding pose of the co-crystallized ligand (**left**) and the corresponding redocked pose (**right**) within the ACC2 active site. The redocked ligand overlaps with the crystallographic conformation and is positioned near Glu539. The reproduced binding pose yielded an RMSD of 1.2 Å relative to the experimental structure, supporting the docking setup for pose prediction.

The docking workflow and representative binding poses of the selected FooDB compounds within the ACC2 carboxyltransferase domain are summarized in Figure 5. The ACC2 structure (PDB ID: 3FF6) is shown with the binding pocket defined by the co-crystallized ligand site. The spatial distribution of the docking region localized predicted ligand interactions within the catalytic domain, and enlarged views showed representative binding conformations of selected compounds within the pocket. These poses were positioned near key residues, including Glu539, consistent with the reference ligand-binding region. Following model-based prioritization, 139,988 FooDB compounds were screened using the trained ACC2 prediction framework. The top 5000 compounds were retained based on ensemble prediction scores, and 200 compounds with predicted activity scores above 0.70 were selected for molecular docking. Docking was performed against the ACC2 carboxyltransferase domain (3FF6) using the binding site defined by the co-crystallized ligand. Redocking of the reference ligand reproduced the experimental binding pose with RMSD of 1.2 Å, supporting the docking setup for pose prediction. From the docking results, six compounds were prioritized based on predicted binding energies and interaction patterns within the ACC2 binding pocket.

Six compounds were retained for further structural analysis based on docking score and ensemble prediction outputs (Table 1). The six compounds were FDB017807 (a vomifoliol triglycoside), FDB023336 (biotinyl-5′-AMP), FDB029596 (lipoyl-GMP), FDB018411 (garcilivin A), FDB017239 (eriojaposide B) and FDB021568 (a dihydroxy-α-ionol apiosyl-glucoside). These are larger, more polar food-derived constituents (molecular weight 516–681 Da) than the antioxidant terpenoids and alkaloids that topped the model ranking, and two of them (biotinyl-5′-AMP and lipoyl-GMP) are nucleotide/cofactor-like conjugates (Table 1 and Table S15). The selected FooDB compounds showed docking scores ranging from −7.47 to −6.65 kcal/mol, compared with −6.21 kcal/mol for the 3FF6 reference ligand. FDB017807 showed the lowest docking score (−7.47 kcal/mol), followed by FDB023336 (−7.24 kcal/mol) and FDB029596 (−7.08 kcal/mol). Ensemble activity probability values for the selected compounds ranged from 0.70 to 0.72, indicating that all six candidates passed the predefined activity-probability threshold used for docking selection. Regression-based predicted activity values ranged from 6.42 to 7.10 pIC50, with FDB029596 showing the highest predicted pIC50 value of 7.10. Pose-level RMSD values varied across the selected compounds, with FDB021568 showing the lowest RMSD value of 0.92 Å, followed by FDB018411 at 2.12 Å and FDB029596 at 2.35 Å. The six compounds were selected using multiple criteria rather than docking rank alone: an ensemble activity probability above the 0.70 screening threshold, favourable and self-consistent docking scores and poses within the carboxyltransferase pocket, recurrent engagement of binding-site residues (notably Glu539), and scaffold diversity to avoid redundant analogues. In practice these criteria were applied jointly rather than as a strict ranking: a compound had to combine a high ensemble activity probability and predicted pIC50 (Table 1) with a plausible, low-RMSD docking pose and recurrent binding-site contacts, and a candidate with a less favourable pose RMSD was retained only when it maintained strong ensemble-model support and consistent Glu539-centred interactions; scaffold diversity was then applied to avoid selecting near-identical analogues. The per-compound metrics underlying these decisions (docking score, pose RMSD, ensemble activity probability and predicted pIC50) are summarized in Table 1. Nearest-neighbour analysis further showed that all six selected compounds have a low maximum Tanimoto similarity to the ACC2 training set (0.16–0.18; Morgan radius 2, 2048 bits) and therefore lie outside the model’s applicability domain (threshold 0.35), as reported in the final column of Table 1 and visualized in Figure S15; their predicted activities should accordingly be treated as extrapolative and exploratory. Together, these docking and ensemble prediction outputs supported the selection of six FooDB compounds for interaction analysis and molecular dynamics simulations.

**Figure 6:**
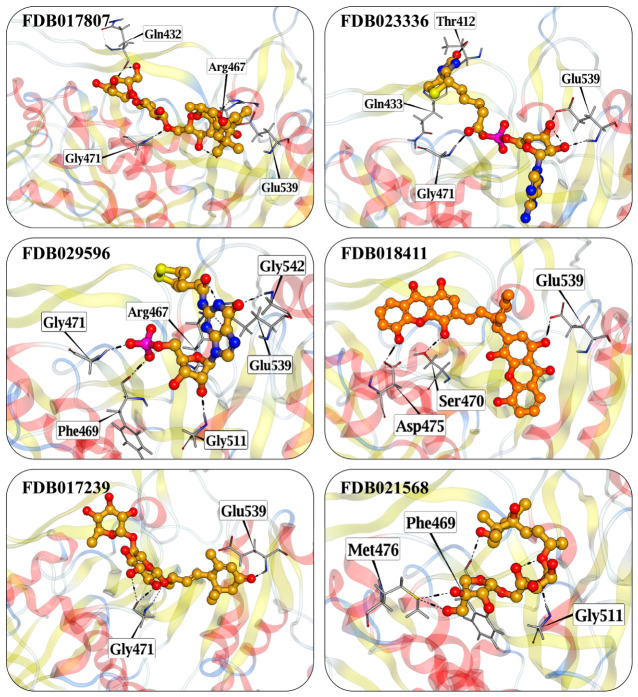
Predicted binding interactions of selected FooDB compounds within the ACC2 active site.

Docking analysis indicated that all six selected FooDB compounds occupied the ACC2 binding pocket and formed predicted interactions with residues lining the catalytic region (Figure 6 and Table S20). A recurrent interaction pattern was observed around Glu539, which was engaged by multiple compounds, including FDB017807, FDB023336, FDB029596 and FDB018411. Gly471 was also frequently contacted across several compounds, indicating a shared contact region within the binding site. FDB017807 and FDB023336 additionally formed predicted interactions with residues in the upper binding region, including Gln432, Gln433, Arg467 and Thr412, suggesting broader engagement across the pocket. FDB029596 showed a wider interaction profile involving Arg467, Phe469, Gly511 and Gly542, consistent with contacts across central and peripheral regions of the binding site. FDB018411 interacted with Asp475 and Ser470, defining an alternative interaction region adjacent to Glu539. FDB021568 showed predicted interactions with Phe469, Met476 and Gly511, whereas FDB017239 primarily engaged Gly471 and Glu539. The recurrence of predicted interactions with Glu539 and neighbouring residues across multiple compounds supports a shared binding region within the ACC2 active site and informed the selection of these compounds for molecular dynamics simulations.

### 2.6. Molecular Dynamics Simulations Assess the Structural Stability of ACC2 Complexes

**Figure 7:**
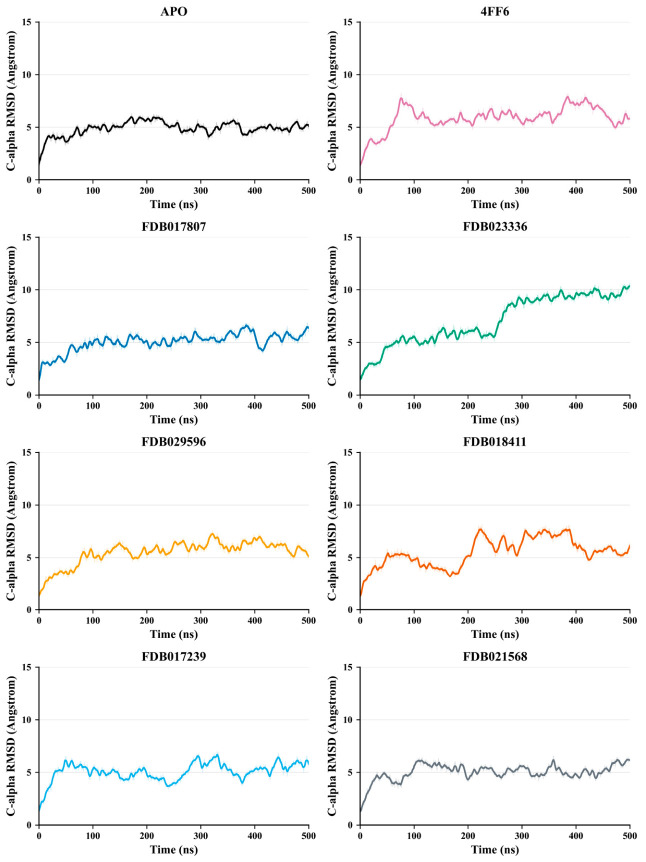
Cα RMSD profiles of apo ACC2, the reference-ligand complex (3FF6) and ACC2 in complex with six selected FooDB compounds over 500 ns simulations.

To examine ACC2 structural stability after ligand binding, 500 ns molecular dynamics simulations were performed for apo ACC2, the reference-ligand complex and the six selected FooDB compound complexes (Figure 7). Cα RMSD profiles were used to monitor global backbone deviations throughout the simulation. Apo ACC2 showed an initial increase in RMSD followed by a relatively steady trajectory across the remaining simulation period. The reference-ligand complex also maintained a defined RMSD profile after early equilibration, with moderate fluctuations during the trajectory. Among the FooDB complexes, FDB017807, FDB029596, FDB017239 and FDB021568 showed comparatively stable RMSD profiles after the early simulation phase. FDB018411 displayed a transient increase during the mid-simulation period before returning to a lower RMSD range. FDB023336 showed a progressive increase in Cα RMSD after approximately 250 ns, indicating greater backbone deviation than the other selected complexes. Overall, RMSD analysis indicated that most ACC2–FooDB complexes maintained stable backbone behaviour over 500 ns, supporting their use in further trajectory-based interaction and binding-energy analyses.

### 2.7. Residue-Wise Flexibility Identifies Localized Dynamic Regions in ACC2 Complexes

**Figure 8:**
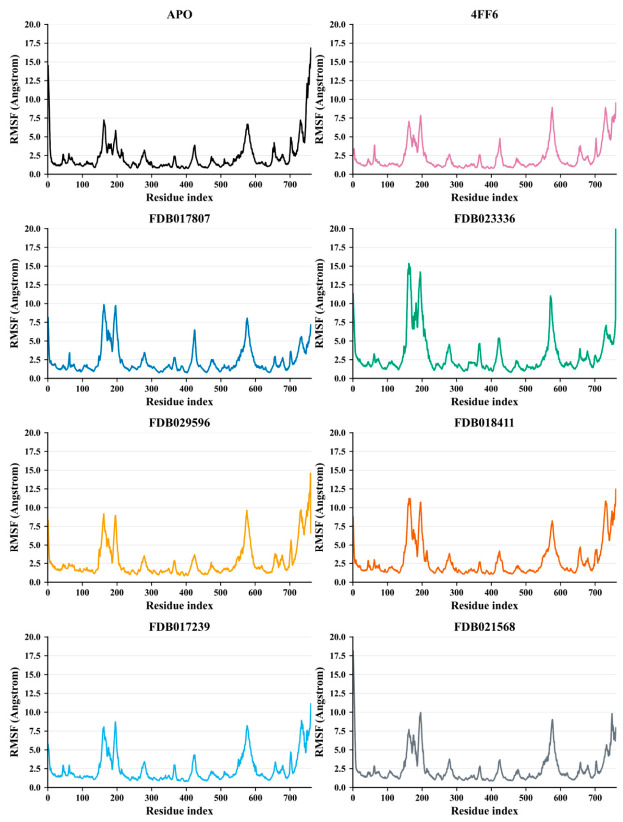
Cα RMSF profiles of apo ACC2, the reference-ligand complex (3FF6) and ACC2 bound to six selected FooDB compounds.

Residue-wise Cα RMSF analysis was performed to examine local flexibility across apo ACC2, the reference-ligand complex and the six FooDB compound complexes during the 500 ns simulations (Figure 8). Across all systems, most residues showed low-to-moderate fluctuations, whereas higher mobility was concentrated in specific protein regions. Apo ACC2 showed localized flexibility around residues 150–200, 570–590 and the terminal regions. A similar pattern was observed for the reference-ligand complex, with prominent fluctuations around residues 170–200, 570–590 and the C-terminal region. Among the FooDB complexes, FDB017807, FDB029596, FDB018411 and FDB017239 showed comparable flexibility peaks around residues 150–200 and 570–590, indicating recurrent dynamic regions across ligand-bound systems. FDB023336 showed stronger local fluctuations than the other FooDB complexes, particularly around residues 160–200, 570–590 and the C-terminal region, consistent with its higher backbone deviation in the RMSD analysis. FDB021568 showed a more restrained RMSF profile across most residues, with localized peaks around residues 170–200, 570–590 and the terminal region. Overall, RMSF analysis indicated that ligand binding did not produce widespread protein destabilization, with flexibility mainly restricted to loop-like and terminal regions of ACC2.

### 2.8. Radius of Gyration Analysis Indicates Maintained Compactness of ACC2 Complexes

The radius of gyration (Rg) was analysed to assess the overall compactness of ACC2 in the apo form, reference-ligand complex and FooDB compound-bound systems over 500 ns simulations (Figure S16). Apo ACC2 maintained a relatively stable Rg profile with minor fluctuations throughout the simulation. The reference-ligand complex showed a comparable Rg range, indicating preserved structural compactness upon ligand binding. Among the FooDB complexes, FDB017807, FDB023336 and FDB029596 exhibited consistent Rg profiles with moderate fluctuations, suggesting stable global folding during the simulation. FDB018411 showed a transient increase followed by a decrease in Rg during the mid-simulation period, indicating temporary expansion and subsequent stabilization. FDB017239 and FDB021568 displayed relatively stable Rg trajectories with minor variations over time. Overall, Rg analysis indicated that ligand binding did not induce major structural expansion or collapse, and the ACC2 complexes retained compactness comparable to the reference-ligand systems throughout the simulation.

### 2.9. Conformational Sampling Across ACC2 Systems

**Figure 9:**
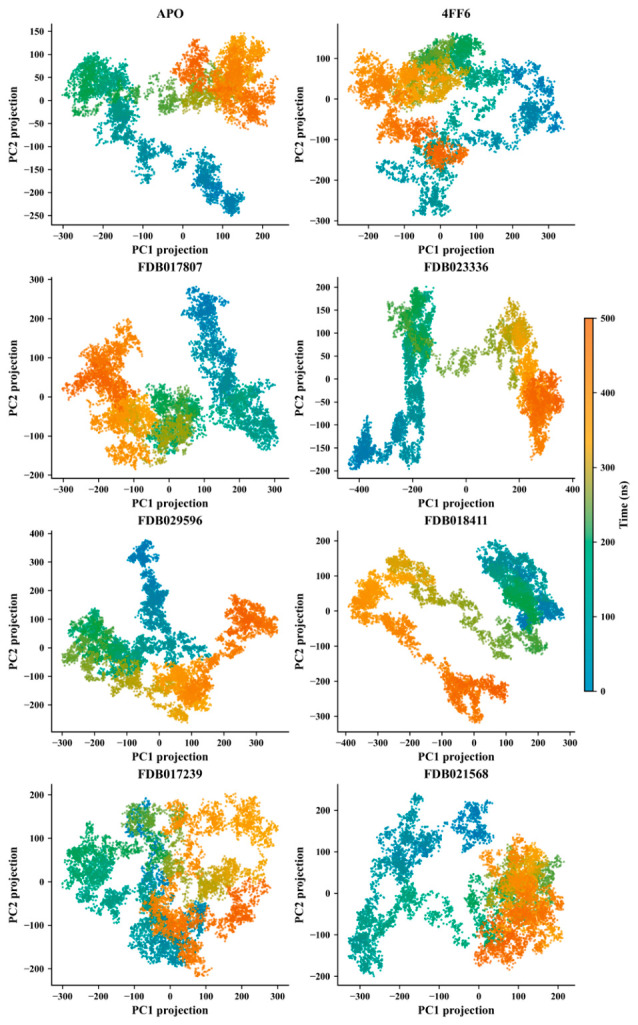
Projection of molecular dynamics trajectories onto the first two principal components (PC1 and PC2) for apo ACC2, the reference-ligand complex (3FF6) and ACC2 bound to six selected FooDB compounds.

Principal component analysis (PCA) was performed on the molecular dynamics trajectories to evaluate large-scale conformational motions of ACC2 in the apo, reference-ligand and FooDB-bound systems (Figure 9). Projection of trajectories onto the first two principal components, PC1 and PC2, revealed distinct clustering patterns corresponding to conformational states sampled during the 500 ns simulations. Apo ACC2 exhibited multiple dispersed clusters, indicating broader conformational sampling with transitions between states throughout the trajectory. The reference-ligand complex showed several partially overlapping clusters, suggesting moderate conformational variability with gradual transitions over time. Among the FooDB complexes, FDB017807 displayed three major clusters corresponding to early (0–150 ns), intermediate (150–350 ns) and late (350–500 ns) simulation phases, indicating progressive conformational transitions. FDB023336 showed two well-separated clusters, with a shift after 250 ns, consistent with its increased structural deviation in the RMSD analysis. FDB029596 exhibited three distinct clusters, reflecting transitions between conformational states across early, mid and late simulation periods. FDB018411 showed two primary clusters, indicating a defined conformational shift during the simulation. By contrast, FDB017239 displayed multiple overlapping clusters, suggesting continuous conformational sampling without sharp transitions. FDB021568 exhibited two main clusters, with a gradual transition between early and late simulation phases. The PCA analysis indicated that ligand binding altered the conformational landscape of ACC2, with most FooDB complexes sampling a limited number of dominant conformational states compared with the broader distribution observed for apo ACC2.

### 2.10. Hydrogen-Bond Analysis Reveals Variable Interaction Stability Among ACC2 Complexes

The number of hydrogen bonds formed between ACC2 and bound ligands was monitored throughout the 500 ns simulations to assess interaction persistence (Figure S17). The reference-ligand complex exhibited a low and intermittent hydrogen-bond profile, with limited interactions observed across the trajectory. Among the FooDB compounds, FDB017807 maintained relatively consistent hydrogen bonding during the early phase, followed by fluctuations and partial loss of interactions during the mid-simulation period, with intermittent recovery at later stages. FDB023336 showed a distinct pattern, with hydrogen bonds forming in the early phase, disappearing during the mid-simulation period and reappearing with sustained interactions after approximately 350 ns. FDB029596 displayed strong hydrogen bonding in the initial phase, followed by a rapid decline, with only sporadic interactions observed for the remainder of the simulation. By contrast, FDB018411 showed a delayed increase in hydrogen-bond formation, with more sustained interactions appearing after approximately 300 ns. FDB017239 exhibited weak and transient hydrogen bonding throughout the simulation, with interactions gradually diminishing toward the later stages. FDB021568 showed moderate and relatively continuous hydrogen bonding, with fluctuations but no complete loss of interactions across the trajectory. Overall, hydrogen-bond analysis indicated that ligand–protein interaction persistence varied across the selected compounds, with some complexes maintaining recurrent interactions whereas others showed transient or phase-dependent bonding behaviour.

Residue-level hydrogen-bond occupancy analysis was used to identify protein residues contributing most frequently to ligand interactions across the simulated ACC2 complexes (Figure S18). The heatmap showed that Glu539 was the most recurrent hydrogen-bonding residue, with detectable occupancy across multiple FooDB complexes and the reference-ligand system. The strongest Glu539 occupancy was observed for FDB018411, while FDB021568 and FDB029596 also retained notable Glu539-mediated contacts. Several additional residues contributed to compound-specific hydrogen-bond patterns. Gly471 showed higher occupancy in the FDB017807 complex and lower occupancy in FDB017239. Phe469 contributed to hydrogen bonding in FDB017807 and FDB021568, whereas Arg467, Ser470, Asp475 and Gln432 were mainly associated with FDB017807. FDB023336 showed contacts involving His363, Leu366, Glu507, Ala434, Lys367 and Thr369, while FDB029596 showed additional occupancy with Glu545 and Arg549. Overall, the residue-occupancy profile indicated that ACC2–ligand hydrogen bonding was concentrated around Glu539, with secondary contacts distributed across neighbouring binding-site residues. This pattern supports Glu539 as a recurrent interaction point within the ACC2 binding pocket during simulation.

### 2.11. Binding Free-Energy Analysis Supports Favourable Ligand–ACC2 Interactions

**Figure 10:**
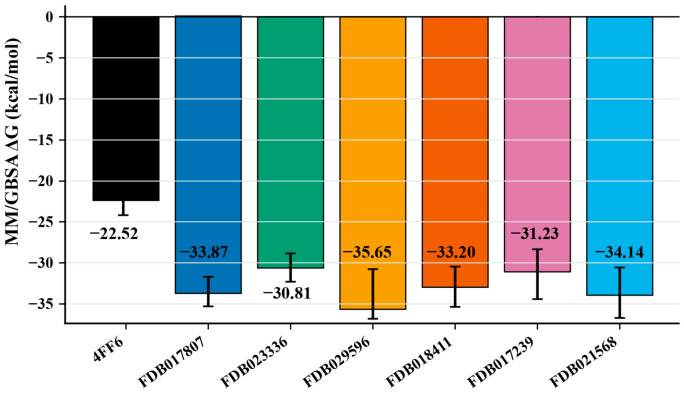
Binding free energies (ΔG) of the reference-ligand complex (3FF6) and ACC2 bound to six selected FooDB compounds calculated using the MM/GBSA method.

Binding free energies of the ACC2–ligand complexes were estimated using the MM/GBSA method to quantify predicted interaction stability over the simulation trajectories (Figure 10). The reference-ligand complex (3FF6) showed a binding free energy of −22.52 kcal/mol and was used as the comparison baseline. All selected FooDB compounds showed more favourable predicted binding free energies than the reference system. Among the selected compounds, FDB029596 showed the most favourable predicted binding free energy (−35.65 kcal/mol), followed by FDB021568 (−34.14 kcal/mol) and FDB017807 (−33.87 kcal/mol). FDB018411 (−33.20 kcal/mol), FDB017239 (−31.23 kcal/mol) and FDB023336 (−30.81 kcal/mol) also showed favourable predicted interaction energies relative to the reference ligand. The distribution of MM/GBSA estimates suggests ligand stabilization within the ACC2 binding pocket across the selected FooDB candidates. These results support the prioritization of the selected compounds as candidate ACC2 binders for biochemical validation.

## 3. Discussion

ACC2 regulates fatty-acid metabolism by controlling malonyl-CoA availability and thereby influencing mitochondrial fatty-acid oxidation [14]. Perturbation of this pathway has been associated with obesity, insulin resistance and related cardiometabolic conditions in which oxidative stress is a recurring feature, supporting ACC2 as a metabolically relevant enzyme for studying lipid-handling mechanisms [27,28]. Although food-derived compounds, and dietary antioxidants in particular, are increasingly recognized as contributors to metabolic regulation, their potential interactions with enzymes involved in lipid handling remain less systematically explored [29]. In this context, dietary compounds should be considered as candidates for nutritional and biochemical evaluation rather than as validated modulators unless experimental evidence is available [30]. Here, we developed an integrated computational nutritional biochemistry workflow combining curated bioactivity data, machine-learning screening, structure-based docking and molecular dynamics simulations to prioritize FooDB compounds with predicted ACC2 interaction potential. The scaffold-split dataset derived from ChEMBL was used to evaluate model performance under structural generalization, reducing the risk of overestimating performance through random-split similarity leakage. The class imbalance observed in the curated dataset reflects the bias toward active compounds in public bioactivity records and supports the need for calibration, threshold selection and applicability-domain analysis before large-scale screening. Benchmarking fingerprint-based, graph-based and graph–Morgan fusion models provided a balanced predictive framework in which regression and classification outputs contributed to compound prioritization. Probability calibration and applicability-domain analysis further constrained downstream screening to predictions with interpretable confidence within the learned chemical space. These steps were important because high predicted activity alone does not establish ACC2 binding, enzymatic modulation or nutritional relevance, particularly for structurally diverse food-derived compounds. Large-scale screening of 139,988 FooDB compounds enabled systematic prioritization of dietary molecules with predicted ACC2 activity propensity. Candidate selection used ensemble mean predictions together with uncertainty estimates to identify compounds in a high-score, low-uncertainty region of the screening landscape. This uncertainty-aware ranking strategy can reduce reliance on single-model scores and provides a more cautious basis for selecting compounds for structure-based evaluation [31]. It should also be emphasized that the six compounds ultimately advanced to docking are chemically distinct from typical ChEMBL ACC2 ligands (being larger, more polar and, in several cases, glycosylated or cofactor-like) and therefore lie toward the boundary of, or outside, the model’s reliable applicability domain; their predicted ACC2 activity consequently carries greater uncertainty than for training-similar compounds, and nearest-neighbour analysis confirms this: all six candidates have a low maximum Morgan–Tanimoto similarity to the ACC2 training set (0.16–0.18; radius 2, 2048 bits) and therefore fall below the 0.35 applicability-domain threshold used here (Table 1 and Figure S15), so their predicted ACC2 activity is an extrapolation beyond the training chemical space and should be regarded as exploratory. Subsequent filtering to 200 high-scoring compounds, followed by docking-based prioritization, provided a multi-stage workflow that progressively refined candidate selection while retaining chemical diversity. Overall, this study establishes a reproducible nutritional biochemistry pipeline for linking bioactivity prediction with structure-based assessment in food-derived chemical space. The resulting candidates should be interpreted as predicted food-derived ACC2 binders that require biochemical validation, including direct binding or enzymatic assays, followed by cellular and nutritional evaluation to determine whether these interactions influence ACC2-associated lipid metabolic regulation [32].

Building on this framework, large-scale application may involve developing extended FooDB models into compound prioritization within a nutritionally relevant chemical space. Screening food-derived compounds expanded the search beyond the curated ACC2 training dataset and introduced broader scaffold and physicochemical diversity. Within this expanded space, ensemble-based ranking prioritized compounds by both predicted activity and agreement across independently trained models. This step is important when screening compounds outside the training distribution, where predictive uncertainty can increase and single-model outputs may be less reliable. The identification of a high-score, low-uncertainty region within the screening landscape provided a rational basis for selecting compounds for structural evaluation. In this study, the filtering strategy retained 5000 top-ranked compounds, followed by refinement to 200 compounds with predicted activity probabilities above 0.70. This multi-stage selection process balanced computational tractability with retention of candidates suitable for downstream docking and simulation analysis. The prioritized FooDB compounds retained chemical diversity rather than converging on a narrow scaffold set. Notably, several of the top-ranked candidates, including the sesquiterpenes caryophyllene, humulene and its epoxides, costunolide and the piperamide alkaloids, are recognized dietary antioxidants with reported anti-inflammatory and redox-modulating activity, aligning the top of the screening ranking with the broader role of antioxidant food compounds in obesity and metabolic disease. This diversity suggests that the predictive model captured multiple structural features associated with ACC2 activity predictions rather than relying on a single chemical motif. Such diversity is useful for subsequent structure-based analyses because it enables assessment of different predicted interaction modes within the ACC2 binding pocket. It also reflects the heterogeneous nature of dietary compounds, which span broad ranges of molecular size, polarity and functional groups. Together, this screening stage linked data-driven prediction with structure-based assessment and enabled systematic selection of candidate ACC2 binders from a large food-derived chemical space. These candidates should be interpreted as computationally prioritized molecules requiring experimental validation, not as confirmed ACC2 modulators.

The antioxidant credentials of the highest-ranked screening classes, which are chemically distinct from the six compounds ultimately advanced to docking, provide additional context for the nutritional relevance of the wider prioritized set. β-Caryophyllene and its oxygenated derivatives, including caryophyllen-15-al, are dietary sesquiterpenes with well-characterized antioxidant and anti-inflammatory activity that has been reported to attenuate oxidative stress, improve lipid handling and ameliorate insulin resistance in models of diet-induced metabolic dysfunction [33]. Costunolide, a sesquiterpene lactone, exhibits free-radical-scavenging, anti-inflammatory and anti-hyperglycaemic properties and modulates lipid metabolism, supporting its candidacy as a redox-active dietary modulator [34]. α-Humulene and its epoxides share the humulane sesquiterpene scaffold and display anti-inflammatory and antioxidant effects relevant to the chronic low-grade inflammation that accompanies obesity [35]. The Piper-derived piperamide alkaloids, represented here by dehydropipernonaline and dipiperamide D, belong to a class with documented antioxidant and lipid-lowering activity, including attenuation of high-fat-diet-induced dyslipidaemia [36]. Although these reported activities are not specific to ACC2, and although these antioxidant classes were the highest-ranked screening hits rather than the compounds ultimately docked, they indicate that the broader FooDB set prioritized by the model is enriched in molecules acting at the intersection of redox balance and lipid metabolism, the axis most relevant to antioxidant-based management of obesity and metabolic disease. The ACC2-binding hypotheses generated here nevertheless remain to be tested directly, and the antioxidant properties cited above should be regarded as supporting context rather than evidence of an ACC2-dependent mechanism. The six compounds carried forward to docking and molecular dynamics were, by contrast, larger and more polar food-derived molecules: glycosylated megastigmanes (a vomifoliol triglycoside, a dihydroxy-α-ionol apiosyl-glucoside and eriojaposide B), an anthraquinone-type dimer (garcilivin A) and two nucleotide/cofactor-like conjugates (biotinyl-5′-AMP and lipoyl-GMP). Docking-based selection therefore favoured molecules capable of extensive hydrogen bonding within the polar carboxyltransferase pocket rather than the smaller antioxidant terpenoids that the model ranked most highly. This distinction has two important implications. First, the favourable MM/GBSA and docking scores of these larger, highly polar and, in two cases, cofactor-derived structures should be interpreted with particular caution, because such molecules are prone to overestimated end-point binding energies and may have limited membrane permeability and restricted access to intracellular, mitochondrially associated ACC2. Second, biotinyl-5′-AMP and lipoyl-GMP are cofactor-biosynthetic intermediates that may represent database-derived endogenous metabolites rather than typical exogenous dietary bioactives, so their nutritional relevance is uncertain and requires explicit experimental confirmation. We therefore present these six molecules only as computationally prioritized binders of the CP-640186 carboxyltransferase pocket, while flagging these physicochemical and provenance caveats; the smaller antioxidant terpenoids and alkaloids ranked most highly by the screen remain chemically plausible dietary candidates for separate structural and nutritional follow-up.

The structure-based stage further refined the FooDB-derived candidate set by assessing whether prioritized compounds could adopt plausible binding orientations within the ACC2 carboxyltransferase domain. Redocking of the co-crystallized ligand reproduced the experimental binding pose with an RMSD of 1.2 Å, supporting the docking setup for pose-level comparison within the 3FF6 binding site. This validation step is important because docking outcomes depend on grid placement, receptor preparation and the ability of the protocol to recover a known ligand orientation [37]. After docking, six FooDB compounds were retained based on predicted binding scores and interaction compatibility with the ACC2 pocket. Their docking scores ranged from −7.47 to −6.65 kcal/mol, compared with −6.21 kcal/mol for the 3FF6 reference ligand, indicating favourable ranking within the same structural environment. These values should be interpreted as docking scores rather than experimental binding affinities. Residue-level inspection provided a structural rationale for this selection. Across the six docked compounds, Glu539 emerged as the most recurrent predicted interaction point, appearing in the interaction profiles of FDB017807, FDB023336, FDB029596 and FDB018411. Gly471 was also repeatedly observed, while Arg467, Phe469, Ser470, Asp475, Gly511, Gly542 and Met476 contributed to compound-specific interaction patterns. This distribution suggests that the selected ligands occupied a shared binding region, with additional contacts extending into neighbouring residue environments. FDB017807 and FDB023336 showed broader predicted engagement across the upper binding region, whereas FDB029596 contacted both central and peripheral residues. FDB018411 formed a more focused interaction pattern adjacent to Glu539, and FDB021568 involved Phe469, Met476 and Gly511. These docking results provided a residue-level basis for advancing the six compounds to molecular dynamics simulations. The interpretation remains limited to predicted structural compatibility. The docking stage should therefore be viewed as a filter for selecting compounds with plausible pocket occupancy and recurring residue contacts, rather than as evidence of experimentally confirmed ACC2 modulation.

Molecular dynamics simulations extended the docking analysis by assessing whether the predicted ACC2–ligand complexes remained structurally coherent over time. Across 500 ns simulations, Cα RMSD profiles showed that most FooDB-bound systems reached comparatively stable backbone behaviour after early equilibration, whereas FDB023336 displayed a progressive increase after approximately 250 ns. This pattern was also reflected in the RMSF analysis, where FDB023336 showed stronger local fluctuations around residues 160–200, 570–590 and the C-terminal region than the other FooDB complexes. By contrast, FDB021568 showed a more restrained residue-flexibility profile across most residues, with localized peaks around residues 170–200, 570–590 and the terminal regions. These observations indicate that compound binding did not produce widespread ACC2 destabilization, with flexibility mainly restricted to likely mobile loop or terminal segments. Radius of gyration analysis provided a complementary measure of global compactness. Apo ACC2, the reference-ligand complex and the FooDB-bound systems retained broadly comparable compactness profiles over the simulation period, indicating that ligand binding did not induce major protein expansion or collapse. FDB018411 showed a temporary increase followed by reduced Rg during the mid-simulation period, consistent with transient structural relaxation rather than persistent destabilization. Principal component analysis further showed that ligand-bound ACC2 systems sampled distinct conformational spaces, with several FooDB complexes occupying two or three dominant clusters rather than the broader conformational spread observed for apo ACC2. This suggests that ligand binding may restrict and redirect large-scale motions of the carboxyltransferase domain, although experimental structural validation is required before functional implications can be assigned [38]. Trajectory-based interaction analysis further supported the compound-specific nature of predicted ACC2 binding. Hydrogen-bond counts varied across complexes, with some ligands showing phase-dependent or intermittent interactions during the simulation. Residue-level occupancy analysis identified Glu539 as the most recurrent hydrogen-bonding residue, with additional ligand-specific contacts involving Gly471, Phe469, Arg467, Ser470, Asp475, Gln432, Gly511 and neighbouring residues. MM/GBSA calculations provided relative binding free-energy estimates across the simulated complexes. All six FooDB compounds showed more favourable estimated binding free energies than the reference-ligand system, with FDB029596, FDB021568 and FDB017807 among the most favourable complexes. Because MM/GBSA is an approximate post-simulation estimator that neglects configurational entropy and can be biased for large, polar or charged solutes, rather than an experimental affinity measurement, these results support relative prioritization of the selected compounds for biochemical testing rather than confirming ACC2 binding or inhibition [39]. Because each complex was simulated in a single 500 ns trajectory and MM/GBSA omits the entropic term, this binding ranking is preliminary and supportive rather than conclusive; the recurrent role of Glu539 is, however, independently supported by the hydrogen-bond occupancy analysis (Figure S17), and per-residue MM/GBSA energy decomposition is identified as a valuable next step to substantiate this interaction hotspot.

This study presents an integrated machine-learning and structure-based workflow for prioritizing food-derived antioxidant compounds with predicted ACC2 interaction potential. The evidence should be read as successive computational layers (machine-learning prioritization, docking-based pose prediction and molecular-dynamics stability assessment), each of which narrows the candidate set but none of which constitutes direct evidence of biological activity. Calibrated ensemble screening, docking validation and 500 ns molecular dynamics simulations enabled the selection of structurally plausible ACC2–ligand complexes from a large FooDB chemical space. Recurrent binding-site contacts, particularly involving Glu539 and neighbouring residues, together with stable trajectory behaviour and favourable MM/GBSA binding free-energy estimates, supported the prioritization of the selected compounds for further evaluation. The main limitations of this work are that all evidence is computational and that no experimental (in vitro or in vivo) wet-laboratory validation was performed, that MM/GBSA and docking provide relative rather than absolute affinity estimates, and that bioavailability, tissue exposure and redox behaviour of the dietary candidates were not assessed. Docking was targeted to the CP-640186 carboxyltransferase-domain pocket of the isolated ACC2 carboxyltransferase domain (PDB 3FF6) using a rigid receptor, so the study represents a pocket-focused screen of this defined site rather than a global or full-length ACC2-binding analysis, and inter-domain or induced-fit effects are not captured; the molecular dynamics simulations partially mitigate this by allowing full receptor and ligand flexibility. Docking performance was assessed by redocking the co-crystallized ligand (RMSD 1.2 Å) rather than by decoy-based enrichment metrics, which were not computed. The molecular dynamics analyses used single 500 ns trajectories per system rather than multiple independent replicates and were initiated from docked poses, so they test pose persistence rather than de novo binding-site discovery. Because ACC1 and ACC2 share a highly conserved carboxyltransferase domain, isoform selectivity cannot be inferred from the present single-isoform docking and simulations. These findings should therefore be interpreted as candidate prioritization rather than evidence of ACC2 modulation. In particular, predicted binding to the carboxyltransferase domain does not by itself establish functional inhibition of ACC2, and the compounds are described throughout as computationally prioritized candidate ACC2 binders rather than as regulators of lipid metabolism. Biochemical inhibition assays, biophysical binding measurements, selectivity profiling and cell-based lipid-metabolism assays, ideally combined with measurement of antioxidant and anti-inflammatory readouts, are required to determine whether the selected food-derived compounds bind ACC2, modulate ACC2 activity or produce measurable metabolic effects relevant to obesity and metabolic disease.

## 4. Materials and Methods

### 4.1. Bioactivity Data Retrieval for ACC2 from ChEMBL 36

Bioactivity records for acetyl-CoA carboxylase 2 (ACC2; ChEMBL target ID: CHEMBL4829) were retrieved from the ChEMBL 36 SQLite database using a target-specific extraction workflow. CHEMBL4829 was used to restrict the query to assays annotated against ACC2. The activity, assay, target, molecule, compound-structure and document tables were queried to retrieve compound identifiers, canonical SMILES, standard activity type, standard relation, standard activity value, standard units, pChEMBL value, assay identifier, assay type, assay confidence score, target annotation and publication year. Records were retained when quantitative standard activity values, accepted activity units and assay-level target-confidence annotations were available. Activity units were restricted to nM and µM. Endpoint records initially included IC50, Ki, Kd and EC50 measurements; downstream modelling was restricted to IC50 values to maintain endpoint consistency. QSAR-ready records were further restricted to exact activity relations, positive standard activity values and compounds with available canonical SMILES. Censored measurements with greater-than or less-than relations were excluded. The curated ACC2 bioactivity dataset was used for pIC50 transformation, compound-level aggregation and machine-learning activity classification.

### 4.2. Data Audit, Structure Standardization and Duplicate Handling

The ACC2 input table was required to contain canonical SMILES, pIC50 and Active columns. SMILES strings were parsed and sanitized, and records with empty, invalid or non-parsable SMILES were removed. Valid molecules were converted to canonical isomeric SMILES. Duplicate records were grouped by canonical isomeric SMILES. Duplicate pIC50 values were aggregated using the maximum value, and duplicate Active labels were aggregated using the maximum value. Exact molecular weight, ring count and heavy-atom count were calculated for molecular quality control. The deduplicated compound table was used for scaffold-based data splitting. Scaffold-based splitting used Bemis–Murcko scaffolds: a scaffold was computed for each compound, and all compounds sharing a scaffold were assigned to the same partition, so that the training, validation and test sets contained disjoint scaffold sets. This scaffold-disjoint partitioning was repeated across three independent random seeds to reduce structural information leakage between the model-development and test partitions.

### 4.3. Molecular Graph Construction

Molecular graphs were generated from the deduplicated ACC2 dataset after joining compound records to scaffold-split assignments by canonical SMILES [40]. Only one-to-one matched records were retained. Each valid SMILES string was parsed and converted into a molecular graph. Atoms were encoded as node features using atomic number, degree, formal charge, hybridization state, aromaticity, ring membership, chirality, implicit hydrogen count, total valence and scaled atomic mass. Bonds were encoded as bidirectional edge features using bond type, conjugation, ring membership, aromatic-bond status and stereochemistry. Node features were represented as 32-bit floating-point tensors, edge indices as 64-bit integer tensors and edge attributes as 32-bit floating-point tensors. Regression labels were encoded from pIC50, and classification labels were encoded from Active. Compounds that could not be converted into valid graph tensors were excluded before model training.

### 4.4. Baseline Fingerprint QSAR Models

Morgan fingerprints [41] were generated from canonical SMILES after joining the deduplicated ACC2 compound table with scaffold-split assignments by canonical SMILES. The merge was performed as a one-to-one mapping between compound records and split labels. Each molecule was parsed and sanitized before fingerprint calculation. Circular Morgan fingerprints were calculated with radius 2 and 2048 bits. Compounds that failed molecular parsing or fingerprint generation were excluded before model fitting. Random forest and histogram-based gradient boosting models were trained as non-graph QSAR baselines. For pIC50 regression, the random forest regressor used 600 estimators, a minimum leaf size of 2, square-root feature sampling and the scaffold-split seed as the random state. The histogram-based gradient boosting regressor used a learning rate of 0.05, 800 boosting iterations and L2 regularization of 1 × 10^−3^. Regression performance was evaluated using root-mean-square error, mean absolute error, coefficient of determination, Pearson correlation and Spearman correlation. For binary ACC2 activity classification, the random forest classifier used 800 estimators, a minimum leaf size of 2, square-root feature sampling, balanced subsampling class weights and the scaffold-split seed as the random state. The histogram-based gradient boosting classifier used a learning rate of 0.05, 800 boosting iterations and L2 regularization of 1 × 10^−3^. Classification probabilities were converted to binary labels using a threshold selected on the validation set by maximizing the Matthews correlation coefficient. This threshold was then fixed and applied unchanged to the test set. Classification performance was evaluated using AUROC, precision–recall area under the curve, Matthews correlation coefficient [42], balanced accuracy, F1 score, Brier score and confusion-matrix counts. Training, validation and test partitions were defined by the scaffold split and were not randomly reassigned during baseline modelling. Test-set metrics were calculated only after model fitting and validation-threshold selection had been completed. These fingerprint-based models served as non-graph baselines for comparison with graph neural-network models.

### 4.5. Graph Neural-Network QSAR Training and Evaluation

The cached molecular graph dataset was loaded with fixed scaffold assignments embedded in the graph metadata. Graphs were separated into training, validation and test subsets according to the predefined scaffold split. Random seeds were fixed during training, and deterministic GPU settings were applied. The test set was not used during hyperparameter selection, early stopping or threshold selection. The graph model used an edge-aware message-passing architecture with atom-level node features and bond-level edge features. Node features were first projected into a learned hidden representation. Message passing was performed using GINE-like blocks, in which source-node and edge features were concatenated, transformed by a multilayer perceptron and aggregated at the destination node by summation. Each block used layer normalization, rectified linear activation and dropout. Graph-level representations were generated using global pooling, with mean, max or concatenated mean–max pooling selected during validation-based hyperparameter tuning. Separate prediction heads were used for regression and classification when both pIC50 and Active labels were modelled. Model training used the AdamW optimizer with validation-based early stopping. Hyperparameters were selected using only the training and validation subsets. Tuned parameters included learning rate, weight decay, dropout, hidden dimension, message-passing depth, batch size, pooling operation, loss function, Huber-loss delta, gradient-clipping threshold, patience and maximum number of epochs. Regression models were optimized using mean-squared-error or Huber loss. Classification models were optimized using binary cross-entropy with logits, with class weighting calculated from the training subset only. For multitask training, regression and classification losses were optimized jointly through a shared graph encoder and task-specific output heads. Validation performance was used as the tuning objective, with negative root-mean-square error used for regression, precision–recall area under the curve used for classification and a combined objective used for multitask modelling. For classification, the decision threshold was selected only from validation-set predictions by maximizing the Matthews correlation coefficient. The selected threshold was applied unchanged to the test set. Regression performance was evaluated using root-mean-square error, mean absolute error, coefficient of determination, Pearson correlation and Spearman correlation. Classification performance was evaluated using AUROC, precision–recall area under the curve, Matthews correlation coefficient, balanced accuracy, F1 score, Brier score and confusion-matrix counts. Final model performance was reported separately for the training, validation and test subsets.

### 4.6. Graph–Morgan Late-Fusion Model Training and Evaluation

The graph cache was loaded with fixed scaffold-split assignments. Morgan fingerprints were generated from the canonical SMILES associated with each graph using radius 2 and 2048 bits. Fingerprints were mapped to graph records by canonical SMILES and used as a parallel molecular representation. The fusion architecture combined an edge-aware graph encoder with a Morgan fingerprint branch. The graph branch used the GINE-like message-passing encoder described above. The fingerprint branch used layer normalization, dropout and fully connected layers to transform the binary Morgan fingerprint into a learned fingerprint embedding. Graph and fingerprint embeddings were concatenated and passed through a fusion multilayer perceptron. Regression and classification outputs were generated from task-specific prediction heads. Model training used AdamW with parameter-group regularization [43]. Weight decay was applied to the graph encoder, fusion layers and output heads, with stronger weight decay applied to the fingerprint-projection branch. Fingerprint bit dropout was applied during training to reduce dependence on individual fingerprint bits. Additional overfitting controls included dropout, gradient clipping, validation-based early stopping, classification label smoothing and exponential moving-average weights for validation selection and final evaluation. Hyperparameter tuning was performed only on the training and validation subsets. Tuned parameters included graph depth, node hidden dimension, graph hidden dimension, pooling operation, fingerprint hidden dimension, fingerprint dropout, fingerprint bit-dropout rate, fusion hidden dimension, head hidden dimension, learning rate, weight decay, batch size, patience, maximum epochs, gradient-clipping threshold, Huber-loss delta, classification label smoothing and exponential moving-average decay. Validation predictions were used to select the best model and, for classification, to lock the Matthews-correlation-optimized probability threshold. The locked threshold was applied to test-set predictions without further adjustment. Regression and classification metrics were calculated using the same evaluation definitions as for the graph-only model. Test-set evaluation was performed only after model selection and was not used for tuning, threshold selection or early stopping.

### 4.7. Multi-Seed Aggregation and Statistical Reporting

Model performance was aggregated across independent scaffold-split seeds. Per-seed metrics from the graph-only and graph–Morgan fusion models were collected for the training, validation and test subsets. Test-set metrics were summarized across seeds as mean ± standard deviation. Paired statistical comparisons between graph-only and graph–Morgan fusion models were performed across matched seeds for each metric. Paired *t*-tests were used for parametric comparisons, and Wilcoxon signed-rank tests were computed as a non-parametric paired alternative when applicable. Multiple comparisons across metrics were corrected using the Benjamini–Hochberg false-discovery-rate procedure. Classification summaries included AUROC, precision–recall area under the curve, Matthews correlation coefficient, balanced accuracy, F1 score and Brier score. Regression summaries included root-mean-square error, mean absolute error, coefficient of determination, Pearson correlation and Spearman correlation.

### 4.8. Validation-Only Probability Calibration

Post hoc probability calibration was fitted only on validation-set classification predictions and then applied to validation and test predictions. Platt scaling was performed by fitting logistic regression to logit-transformed uncalibrated probabilities [44]. Isotonic regression was used as a non-parametric alternative when specified. Records with invalid labels or missing probabilities were excluded from calibration fitting. Classification thresholds were selected on calibrated validation probabilities by maximizing the Matthews correlation coefficient and then applied unchanged to calibrated test probabilities. Calibration performance was evaluated using AUROC, precision–recall area under the curve, Matthews correlation coefficient, balanced accuracy, F1 score, Brier score, expected calibration error and confusion-matrix counts. Reliability diagrams, probability-distribution plots, receiver operating characteristic curves, precision–recall curves and confusion matrices were generated before and after calibration.

### 4.9. Interpretability, Applicability-Domain and Error Analysis

The selected graph–Morgan fusion model and corresponding graph cache were loaded for each seed. Morgan fingerprints were generated from canonical SMILES using radius 2 and 2048 bits. The applicability domain was estimated for each test compound as the maximum Tanimoto similarity between its Morgan fingerprint and the Morgan fingerprints of compounds in the training set. Compounds with maximum training-set similarity greater than or equal to the predefined threshold were assigned inside the applicability domain. Test-set performance was additionally stratified across quantile-based similarity bins to assess model behaviour as a function of chemical similarity to the training distribution. Latent chemical-space representations were extracted from the fusion embedding layer and projected into two dimensions using UMAP, when available, or principal component analysis otherwise. Projected test compounds were visualized according to maximum Tanimoto similarity [45], predicted classification probability and predicted regression activity. Classification error analysis assigned test compounds as correct predictions, false positives or false negatives using the validation-locked classification threshold. Regression error analysis ranked compounds by absolute prediction error. Atom-level attribution was calculated for selected test compounds using gradient-weighted input attribution with respect to node features. Atom importance values were normalized to the range 0–1 and rendered on molecular structures. Compound labels were taken from PubChem metadata when available and otherwise from canonical SMILES.

### 4.10. FooDB Screening-Library Preparation

FooDB [26] compounds were screened as candidate dietary bioactives using the trained ACC2 graph–Morgan fusion models. The FooDB input table was required to contain compound identifiers and a SMILES field. SMILES strings were parsed and sanitized with RDKit, and invalid, empty or non-parsable structures were excluded. Salts and mixtures were reduced to the largest molecular fragment when fragment standardization was applied. Charge neutralization was not applied by default, preserving charged food-derived structures unless the same operation had been used during model training. Valid structures were converted to canonical isomeric SMILES, and duplicate compounds were collapsed by canonical SMILES. The refined FooDB library was used for graph generation and fingerprint-based inference.

### 4.11. FooDB Graph and Fingerprint Generation

Molecular graphs were generated from the refined FooDB structures using the atom and bond featurization scheme used for ACC2 model training. Atom features comprised atomic number, degree, formal charge, hybridization state, aromaticity, ring membership, chirality, total hydrogen count, total valence and scaled atomic mass. Bond features comprised bond type, conjugation, ring membership, aromatic-bond status and stereochemistry. Bonds were represented as bidirectional edges. Molecules with invalid structures, zero atoms or atom counts above the predefined maximum were excluded. Compound identifiers, names and canonical SMILES were retained as graph-level metadata. Morgan fingerprints were generated from the same canonical SMILES used for graph construction. Circular Morgan fingerprints were calculated with radius 2 and 2048 bits. Fingerprint rows were aligned to the graph order so that each FooDB compound had matched graph and fingerprint representations during fusion-model inference. A screening index containing compound identifiers, names and canonical SMILES was used for downstream ranking and traceability.

### 4.12. Ensemble Screening and Ranking of FooDB Compounds

Trained graph–Morgan fusion checkpoints from independent scaffold-split seeds were used as an ensemble for FooDB screening. FooDB graph tensors and Morgan fingerprints were processed in batches. For classification, each model generated a predicted ACC2 activity probability for every FooDB compound, and the ensemble score was calculated as the mean predicted probability across seeds. Ensemble uncertainty was calculated as the standard deviation of predicted probabilities across seeds. For regression, predicted activity was calculated as the mean predicted value across seeds, with ensemble standard deviation used as the uncertainty estimate. Compounds were ranked by ensemble mean score, with lower ensemble uncertainty used as the secondary ranking criterion. The complete scored FooDB library and the top-ranked subset were retained for post-screening analysis.

### 4.13. Post-Screening Analysis of FooDB-Ranked Compounds

Score distributions were calculated for the full FooDB screening set, and uncertainty distributions were calculated from ensemble standard deviations. Top-ranked FooDB compounds were binned by prediction score and uncertainty. A high-confidence subset was defined as compounds with high ensemble scores and low ensemble uncertainty according to predefined quantile thresholds. Bemis–Murcko scaffolds were generated to assess chemical diversity among the top-ranked compounds. Scaffold clusters were summarized, and scaffold representatives were selected by retaining the highest-scoring compound from each scaffold group. Physicochemical properties were calculated for top-ranked FooDB compounds, including molecular weight, LogP, hydrogen-bond donors, hydrogen-bond acceptors, topological polar surface area, rotatable bonds, ring count and heavy-atom count. When the ACC2 training set was supplied, each FooDB hit was compared with training compounds using maximum Morgan Tanimoto similarity to identify close analogues and chemically distinct candidates.

### 4.14. ACC2 Crystal Structure Selection

The top 200 FooDB-ranked compounds were selected for molecular docking against the ACC2 carboxyltransferase domain. The crystal structure of the human ACC2 carboxyltransferase domain in complex with CP-640186 was obtained from the Protein Data Bank using PDB ID 3FF6 [46]. The deposited structure corresponds to the human acetyl-CoA carboxylase 2 carboxyltransferase domain bound to CP-640186 and was determined by X-ray diffraction. The co-crystallized CP-640186 ligand was used to define the docking site. Protein preparation was performed using freely available molecular-modelling tools. Crystallographic water molecules and non-relevant heteroatoms were removed, while the co-crystallized ligand was retained during binding-site definition. Missing residues and unresolved loop regions were modelled where required. N- and C-terminal groups were assigned appropriate terminal charges, and hydrogen atoms were added according to the selected protonation state. Bond orders, atom types and formal charges were checked after hydrogen addition. The prepared receptor structure was locally energy-minimized to relieve steric clashes introduced during preparation while preserving the crystallographic binding-site geometry. The co-crystallized ligand was then removed from the prepared receptor before docking of FooDB compounds.

### 4.15. Ligand Preparation

The top 200 FooDB compounds were prepared from their canonical SMILES structures. Molecular structures were converted into three-dimensional conformations, hydrogen atoms were added, and atom types and formal charges were assigned. Each ligand was geometry-optimized before docking. Ligand structures were converted into AutoDock-compatible PDBQT format, with rotatable bonds assigned for flexible-ligand docking. Compounds that failed structure conversion, hydrogen addition, geometry optimization or PDBQT preparation were excluded from docking.

### 4.16. Molecular Docking Using AutoDock Vina

Molecular docking was performed using AutoDock Vina 1.2.5 [47] with the prepared ACC2 carboxyltransferase-domain receptor and prepared FooDB ligand structures. The docking grid was centred on the binding site occupied by the co-crystallized CP-640186 ligand in PDB 3FF6. A cubic search box of 40 × 40 × 40 Å was used to cover the CP-640186 binding pocket and adjacent ligand-accessible residues. The receptor was treated as rigid, and ligands were treated as flexible. Docking was performed using the Vina scoring function, and multiple binding poses were generated for each ligand. The best-ranked pose for each compound was selected according to the lowest predicted binding energy. Docked poses were retained for interaction inspection and ranking of FooDB candidates.

### 4.17. Docking-Pose Analysis

Docked poses of the top-ranked FooDB compounds were analysed within the ACC2 CP-640186 binding pocket. Binding modes were inspected relative to the co-crystallized ligand position. Protein–ligand interactions were evaluated for hydrogen bonding, hydrophobic contacts, aromatic interactions and electrostatic contacts. Compounds were prioritized using docking score, binding-pose consistency within the CP-640186 site and interaction compatibility with the ACC2 carboxyltransferase-domain binding pocket.

### 4.18. Molecular Dynamics Simulations

Molecular dynamics simulations were performed for apo ACC2, the ACC2–CP-640186 complex from PDB 3FF6 as the positive-control system, and six selected ACC2–FooDB candidate complexes. Prepared ACC2 structures and ligand-bound complexes were used as starting coordinates. Protein parameters were assigned using the AMBER24 [48] ff19SB force field [49]. Ligand parameters were generated with GAFF2 [50], and ligand partial charges were assigned before system assembly. Systems were prepared with LEaP, solvated in an OPC water box with a minimum solvent buffer of 12 Å around the solute, and neutralized by adding counterions. Long-range electrostatics were treated using the particle-mesh Ewald method, and a 10 Å non-bonded cutoff was applied for short-range interactions. Each system was minimized in two stages before equilibration. In the first stage, positional restraints were applied to protein and ligand heavy atoms while solvent molecules and ions were relaxed. In the second stage, restraints were removed and the complete system was minimized. Energy minimization used steepest descent [51] followed by conjugate-gradient minimization [52], with 50,000 steepest-descent steps and 20,000 conjugate-gradient cycles. Systems were then heated gradually over 500 ps under positional restraints, followed by density adjustment and equilibration. A 10 ns equilibration phase was performed before production simulation. Production molecular dynamics simulations were performed with PMEMD for 500 ns per system. Bonds involving hydrogen atoms were constrained using SHAKE, allowing a 2 fs integration time step. Temperature was controlled using a Langevin thermostat, and pressure was maintained under periodic boundary conditions in the isothermal–isobaric ensemble after density adjustment. Coordinates were stored every 50 ps for trajectory analysis. The same simulation protocol was applied to apo ACC2, the CP-640186 positive-control complex and the six FooDB candidate complexes to enable direct comparison of structural stability and ligand-binding behaviour.

### 4.19. Post-Simulation Trajectory Analysis

Production trajectories were analysed after removal of periodic imaging artefacts using CPPTRAJ [53]. Each trajectory was re-centred and imaged into the primary simulation box before structural measurements were calculated. Trajectory frames were fitted to an equilibrated reference structure using Cα atoms to remove whole-system translation and rotation. The same alignment procedure was applied to apo ACC2, the CP-640186 positive-control complex and the six FooDB candidate complexes before comparative analysis.

#### 4.19.1. Protein Stability and Flexibility Analysis

Protein structural stability was evaluated from Cα root-mean-square deviation calculated across the 500 ns production trajectory using the equilibrated structure as the reference. Residue-wise flexibility was assessed from Cα root-mean-square fluctuation values calculated over the aligned production frames. Protein compactness was monitored using the radius of gyration throughout the trajectory.

#### 4.19.2. Essential Dynamics and Conformational-Space Analysis

Dominant protein motions were examined using principal component analysis of aligned Cα positional coordinates. Positional covariance matrices were generated from the production trajectories and decomposed to identify the principal modes contributing most strongly to large-scale conformational variation. The first two principal components were used to compare conformational sampling among simulated systems. Backbone-based clustering was performed on regularly sampled trajectory frames using k-means clustering. Cluster representatives were selected as the structures closest to the centroid of the most populated conformational clusters.

#### 4.19.3. Protein–Ligand Interaction Persistence

Protein–ligand hydrogen bonds were quantified across production trajectories using geometric donor–acceptor and angular criteria. A hydrogen bond was assigned when the donor–acceptor distance was ≤3.5 Å and the donor–hydrogen–acceptor angle was ≥120°. Hydrogen-bond occupancy was calculated as the fraction of analysed frames in which each interaction was present. Persistent interactions were distinguished from short-lived contacts using occupancy across equilibrated trajectory frames. Binding-site contacts were further inspected to compare interaction stability between the CP-640186 positive-control complex and the FooDB candidate complexes.

#### 4.19.4. MM/GBSA Binding Free-Energy Estimation

Binding free energies were estimated from snapshots extracted from the final 100 ns of each production trajectory. This interval was selected after inspection of backbone stability and ligand-interaction profiles. Binding free energy was calculated as the difference between the free energy of the complex and the summed free energies of the separated receptor and ligand:

ΔG_bind_ = G_complex_ − (G_receptor_ + G_ligand_)

The total free energy was decomposed into molecular-mechanics and solvation terms:

ΔG_bind_ = ΔE_MM_ + ΔG_solv_ − TΔS

where ΔE_MM_ is the molecular-mechanics energy, comprising bonded (ΔE_internal_), van der Waals (ΔE_vdW_) and electrostatic (ΔE_ele_) terms. ΔG_solv_ is the solvation free energy, partitioned into a polar contribution (ΔG_GB_) estimated with the generalized Born model and a nonpolar contribution (ΔG_SA_) derived from the solvent-accessible surface area. The entropic term (−TΔS) was not included. Energy values were reported in kcal/mol.

### 4.20. Data Analysis and Software

All computational analyses were performed using Python 3.11 [54]. Molecular parsing, canonicalization, fingerprint generation, scaffold analysis and physicochemical-property calculations were performed using RDKit 2026.3.1 [55]. Neural-network models were implemented and evaluated using PyTorch 2.11.0 [56]. Data handling and numerical analysis used pandas 3.0.2 [57] and NumPy 2.4.1 [58]. Machine-learning metrics, calibration analysis and statistical utilities used scikit-learn 1.8.0 [58] and SciPy 1.16.3 [59]. Figures were generated using Matplotlib 3.10.9 [60].

## 5. Conclusions

This study developed an integrated computational workflow to prioritize food-derived antioxidant compounds with predicted ACC2 interaction potential, a target positioned at the intersection of lipid metabolism, insulin resistance and oxidative stress in obesity and metabolic disease. Curated ACC2 bioactivity data were used to train and evaluate scaffold-split machine-learning models, and calibrated ensemble predictions were applied to 139,988 FooDB compounds. Subsequent filtering, molecular docking and 500 ns molecular dynamics simulations prioritized six FooDB compounds with favourable predicted binding profiles within the ACC2 carboxyltransferase domain. The selected compounds (larger, polar food-derived molecules including glycosides and two nucleotide/cofactor-like conjugates, chemically distinct from the antioxidant terpenoids and alkaloids that topped the screening ranking) showed predicted engagement of the ACC2 binding pocket, with recurrent interactions involving Glu539 and neighbouring residues. Molecular dynamics analyses indicated that most ACC2–ligand complexes maintained backbone stability, localized residue flexibility and preserved compactness, while MM/GBSA analysis estimated favourable binding free energies relative to the reference ligand. Together, these findings provide a ranked set of food-derived candidate ACC2 binders and a structure-supported basis for future ACC2-focused nutritional biochemistry studies on antioxidants in the management of obesity and metabolic disease.

## Figures and Tables

**Table 1:** Docking scores and ensemble prediction metrics for top-ranked FooDB compounds targeting ACC2. The final column reports each compound’s maximum Morgan–Tanimoto similarity to the ACC2 training set and its applicability-domain (AD) status (AD threshold 0.35).

Compound	2D Structure	Docking Score (kcal/mol)	RMSD (Å)	Ensemble Activity Probability (Mean)	Predicted Activity (pIC50, Mean)	Max Tanimoto to Training Set (AD Status)
FDB017807	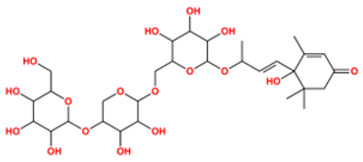	−7.47	4.98	0.71	6.48	0.18 (outside)
FDB023336	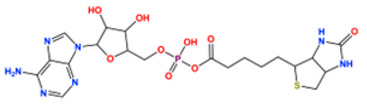	−7.24	3.67	0.71	6.86	0.16 (outside)
FDB029596	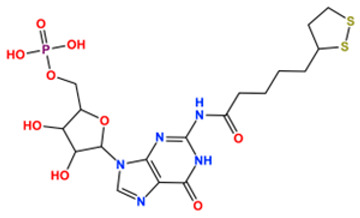	−7.08	2.35	0.70	7.10	0.18 (outside)
FDB018411	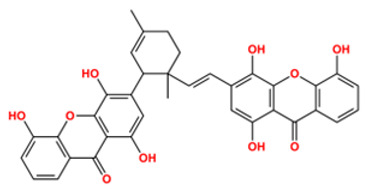	−6.94	2.12	0.72	6.79	0.18 (outside)
FDB017239	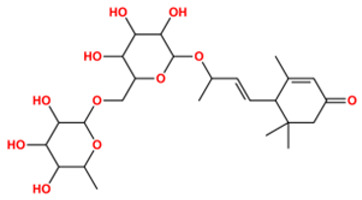	−6.81	3.14	0.72	6.62	0.17 (outside)
FDB021568	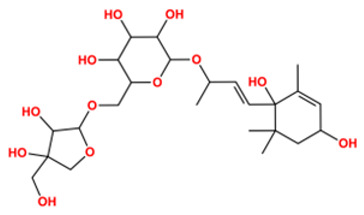	−6.65	0.92	0.71	6.42	0.18 (outside)
3FF6 (reference)	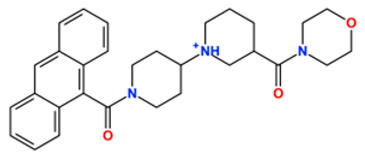	−6.21	–	–	–	–

## Data Availability

All scripts and code used in this study, including the graph neural-network framework and associated data processing for ACC2 structure–function analysis, are publicly available at https://github.com/nalznedy/ACC2-Enzyme-GNN-Pipeline (accessed on 27 December 2025). ACC2 bioactivity data were obtained from ChEMBL 36, food-compound structures from FooDB, and the ACC2 carboxyltransferase-domain crystal structure from the Protein Data Bank (PDB ID: 3FF6).

## Supplementary Materials

The following supporting information can be downloaded at: https://www.mdpi.com/article/10.3390/ph19071123/s1. Figure S1: Scaffold-split stability and molecular graph-size distributions across independent seeds; Figure S2: Per-seed random forest regression diagnostics for ACC2 pIC50 prediction; Figure S3: Per-seed histogram gradient boosting regression diagnostics for ACC2 pIC50 prediction; Figure S4: Training behaviour and per-seed regression diagnostics for the graph neural-network model; Figure S5: Training behaviour and per-seed regression diagnostics for the graph–Morgan fusion model; Figure S6: Per-seed random forest classification diagnostics for ACC2 activity prediction; Figure S7: Per-seed histogram gradient boosting classification diagnostics for ACC2 activity prediction; Figure S8: Per-seed graph neural-network classification diagnostics for ACC2 activity prediction; Figure S9: Per-seed graph–Morgan fusion classification diagnostics for ACC2 activity prediction; Figure S10: Validation-based Platt calibration of graph–Morgan fusion classification probabilities; Figure S11: Test-set similarity to the ACC2 training set and chemical-space distribution across scaffold-split seeds; Figure S12: Chemical-space projections coloured by ACC2 model predictions; Figure S13: Classification and regression performance across nearest-neighbour similarity bins; Figure S14: Representative atom-level attribution maps from the graph–Morgan fusion model; Figure S15: Applicability-domain status (maximum Morgan–Tanimoto similarity to the ACC2 training set) of the six selected compounds; Figure S16: Radius of gyration (Rg) trajectories of apo ACC2, the reference-ligand complex (3FF6) and ACC2 bound to six selected FooDB compounds; Figure S17: Number of hydrogen bonds between ACC2 and the reference ligand (3FF6) and six selected FooDB compounds over 500 ns simulations; Figure S18: Hydrogen-bond residue-occupancy heatmap for the reference-ligand complex and six FooDB-bound ACC2 systems; Table S1: Per-seed scaffold-split composition of the curated ACC2 dataset; Table S2: Mean and standard deviation of scaffold-split statistics across independent seeds; Table S3: Global Bemis–Murcko scaffold distribution in the curated ACC2 dataset; Table S4: Molecular graph composition and label coverage for scaffold-split seed 1; Table S5: Molecular graph composition and label coverage for scaffold-split seed 2; Table S6: Molecular graph composition and label coverage for scaffold-split seed 3; Table S7: Full regression and classification performance metrics for all models, splits and seeds; Table S8: Paired statistical comparisons of test-set model performance across scaffold-split seeds; Table S9: Model performance within the ACC2 applicability domain across scaffold-split seeds; Table S10: ACC2 model performance stratified by chemical similarity to the training set; Table S11: Test-set chemical-space embeddings and nearest-neighbour similarity annotations; Table S12: Top-ranked FooDB compounds from ensemble ACC2 activity screening; Table S13: High-confidence FooDB candidates selected by ensemble score and uncertainty filtering; Table S14: Scaffold-diverse representatives from FooDB ensemble screening; Table S15: Physicochemical properties of top-ranked FooDB screening candidates; Table S16: Hard false-positive compounds identified from ACC2 classification error analysis; Table S17: Hard false-negative compounds identified from ACC2 classification error analysis; Table S18: Test compounds with the largest absolute pIC50 prediction errors; Table S19: Atom-attribution index for representative compounds used in model interpretation; Table S20: Protein–ligand interaction profiles for docked FooDB compounds in ACC2.

## Abbreviations

The following abbreviations are used in this manuscript:

ACC2	Acetyl-CoA carboxylase 2
AUROC	Area under the receiver operating characteristic curve
GAFF2	General AMBER force field 2
GNN	Graph neural network
MCC	Matthews correlation coefficient
MD	Molecular dynamics
MM/GBSA	Molecular mechanics/generalized Born surface area
PCA	Principal component analysis
PDB	Protein Data Bank
QSAR	Quantitative structure–activity relationship
Rg	Radius of gyration
RMSD	Root-mean-square deviation
RMSF	Root-mean-square fluctuation
TPSA	Topological polar surface area
