# Supplementary material for: A Deep Learning Framework for the Discovery of Natural-Product Candidate Binders of Acetyl-CoA Carboxylase 2 (ACC2) with Potential Relevance to Cardiometabolic Lipid Metabolism
Source: Pharmaceuticals (Basel). 2026 Jul 21;19(7):1123. doi: 10.3390/ph19071123 (PMC13414482; doi:10.3390/ph19071123)
Supplement: Supplementary file 1 [file pharmaceuticals-19-01123-s001.zip › Supplementary Figures_revised.pdf]

Supplementary Figures

A Deep Learning Framework for the Discovery of Natu-ral-Product Candidate Binders of Acetyl-CoA Carboxylase 2 (ACC2) with Potential Relevance to Cardiometabolic Lipid Metabolism

Nada A. Alzunaidy [1*]

[1] Department of Food Science and Human Nutrition, College of Agriculture and Food, Qassim University, Buraydah 51452, Saudi Arabia.

[*] Corresponding author: E-mail address: n.alznedy@qu.edu.sa

Table of Contents

Figure S1. Scaffold-split stability and molecular graph-size distributions across independent seeds. **A**, Train, validation and test split sizes across three independent scaffold-split seeds. **B–D**, Distribution of atom counts per molecular graph in the train, validation and test partitions for seeds 1–3. **E–G**, Distribution of directed-edge counts per molecular graph in the train, validation and test partitions for seeds 1–3. The comparable atom-count and edge-count profiles indicate consistent molecular graph construction across scaffold-split partitions....................................... 4

Figure S2. Per-seed random forest regression diagnostics for ACC2 pIC50 prediction. **A–C**, Predicted versus observed pIC50 values for the random forest Morgan-fingerprint regression model across scaffold-split seeds 1–3. **D–F**, Corresponding test-set residual distributions for each seed. Dashed lines in the predicted-versus-observed plots indicate the ideal prediction line, and dashed lines in the residual plots indicate zero residual. 5

Figure S3. Per-seed histogram gradient boosting regression diagnostics for ACC2 pIC50 prediction. **A–C**, Predicted versus observed pIC50 values for the histogram gradient boosting Morgan-fingerprint regression model across scaffold-split seeds 1–3. **D–F**, Corresponding test-set residual distributions for each seed. Dashed lines in the predicted-versus-observed plots indicate the ideal prediction line, and dashed lines in the residual plots indicate zero residual.................. 5

Figure S4. Training behaviour and per-seed regression diagnostics for the graph neural-network model. **A–C**, Training and validation loss curves for the graph neural-network regression model across scaffold-split seeds 1–3. **D–F**, Predicted versus observed pIC50 values on the test set for

each seed. **G–I**, Corresponding test-set residual distributions. These diagnostics show model convergence and residual behaviour across independent scaffold splits. 6
Figure S5. Training behaviour and per-seed regression diagnostics for the graph–Morgan fusion model. **A–C**, Training and validation loss curves for the graph–Morgan fusion regression model across scaffold-split seeds 1–3. **D–F**, Predicted versus observed pIC50 values on the test set for each seed. **G–I**, Corresponding test-set residual distributions. The plots summarize convergence, prediction agreement and residual structure for the late-fusion molecular representation............ 7
Figure S6. Per-seed random forest classification diagnostics for ACC2 activity prediction. **A–C**, Test receiver operating characteristic curves for the Morgan-fingerprint random forest classifier across scaffold-split seeds 1–3. **D–F**, Corresponding precision–recall curves. **G–I**, Test-set calibration plots comparing predicted probabilities with observed positive fractions for each seed. 8
Figure S7. Per-seed histogram gradient boosting classification diagnostics for ACC2 activity prediction. **A–C**, Test receiver operating characteristic curves for the Morgan-fingerprint histogram gradient boosting classifier across scaffold-split seeds 1–3. **D–F**, Corresponding precision–recall curves. **G–I**, Test-set calibration plots for each seed.. 9
Figure S8. Per-seed graph neural-network classification diagnostics for ACC2 activity prediction. **A–C**, Test receiver operating characteristic curves for the graph neural-network classifier across scaffold-split seeds 1–3. **D–F**, Corresponding precision–recall curves. **G–I**, Test-set calibration plots for each seed.. 10
Figure S9. Per-seed graph–Morgan fusion classification diagnostics for ACC2 activity prediction. **A–C**, Train and test receiver operating characteristic curves for the graph–Morgan fusion classifier across scaffold-split seeds 1–3. **D–F**, Test-set precision–recall curves for each seed. **G–I**, Test-set calibration plots showing the relationship between predicted probabilities and observed positive fractions.. 11
Figure S10. Validation-based Platt calibration of graph–Morgan fusion classification probabilities**.** **A–C**, Validation-set reliability diagrams before and after Platt scaling across scaffold-split seeds 1–3. **D–F**, Validation-set predicted-probability distributions before and after calibration. **G–I**, Test-set receiver operating characteristic curves before and after Platt scaling. Calibration changed the probability scale but preserved test-set discrimination... 12
Figure S11. Test-set similarity to the ACC2 training set and chemical-space distribution across scaffold-split seeds**.** **A–C**, Distributions of maximum Morgan Tanimoto similarity between test compounds and training compounds for scaffold-split seeds 1–3. **D–F**, Two-dimensional test-set chemical-space projections coloured by maximum Tanimoto similarity to the training set for each seed. 13
Figure S12. Chemical-space projections coloured by ACC2 model predictions. **A–C**, Two-dimensional test-set chemical-space projections coloured by predicted ACC2 activity probability for scaffold-split seeds 1–3. **D–F**, Corresponding projections coloured by predicted pIC50 values. These plots show how classification and regression outputs vary across the scaffold-held-out chemical space. 13
Figure S13. Classification and regression performance across nearest-neighbour similarity bins**.** **A–C**, Precision–recall area under the curve across maximum Tanimoto similarity bins for scaffold-split seeds 1–3. **D–F**, Root-mean-square error across the same similarity bins. Higher similarity to training compounds was generally associated with stronger classification performance and lower regression error.. 14

Figure S14. Representative atom-level attribution maps from the graph–Morgan fusion model. Atom-level attribution maps are shown for representative test compounds selected from the interpretability analysis. Highlighted atoms indicate molecular regions contributing to the model output for the displayed prediction task. These maps describe model-attribution patterns and do not establish experimentally validated ACC2 binding interactions. 16
Figure 15. Applicability-domain status of the six selected FooDB compounds. Bars show the maximum Morgan–Tanimoto similarity (radius 2, 2048 bits) of each compound to the ACC2 training set; the dashed line marks the applicability-domain (AD) threshold of 0.35. All six compounds fall below the threshold and therefore lie outside the model's reliable applicability domain, indicating that their predicted ACC2 activities are extrapolations beyond the training chemical space. 16
Figure S16. Radius of gyration (Rg) trajectories of apo ACC2, the reference-ligand complex (3FF6), and ACC2 bound to six selected FooDB compounds over 500 ns simulations............... 18
Figure S17. Number of hydrogen bonds formed between ACC2 and the reference ligand (3FF6) and six selected FooDB compounds over 500 ns molecular dynamics simulations..................... 20
Figure S18. Heatmap showing hydrogen bond residue occupancy for the reference-ligand complex and six FooDB-bound ACC2 systems during molecular dynamics simulations. Rows represent ACC2 residues involved in ligand hydrogen bonding, and columns represent simulated systems. The colour scale indicates summed contact occupancy, with higher values corresponding to more persistent hydrogen bond interactions during the trajectory... 20

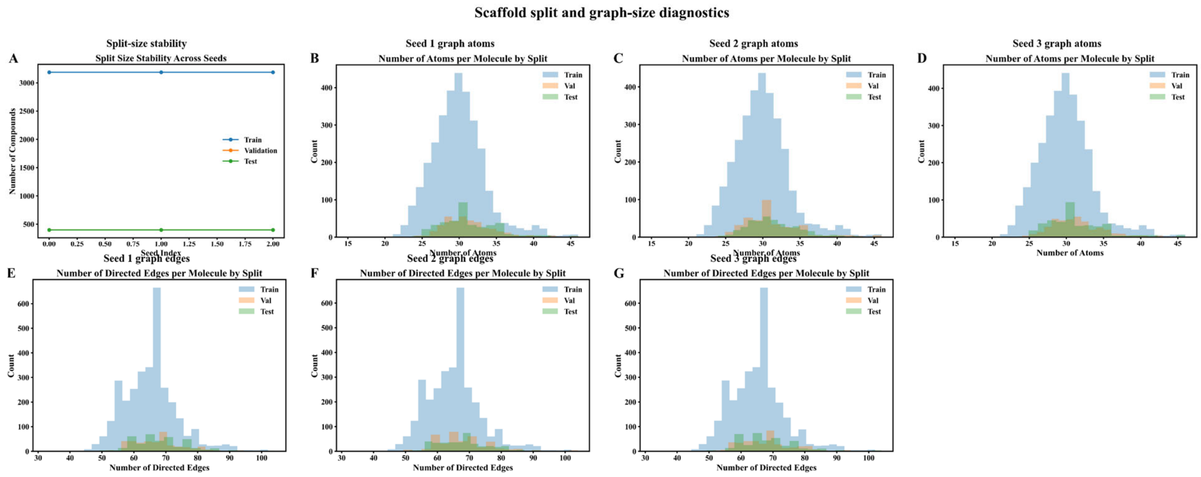

Figure S1. Scaffold-split stability and molecular graph-size distributions across independent seeds. **A**, Train, validation and test split sizes across three independent scaffold-split seeds. **B–D**, Distribution of atom counts per molecular graph in the train, validation and test partitions for seeds 1–3. **E–G**, Distribution of directed-edge counts per molecular graph in the train, validation and test partitions for seeds 1–3. The comparable atom-count and edge-count profiles indicate consistent molecular graph construction across scaffold-split partitions.

Random forest regression diagnostics

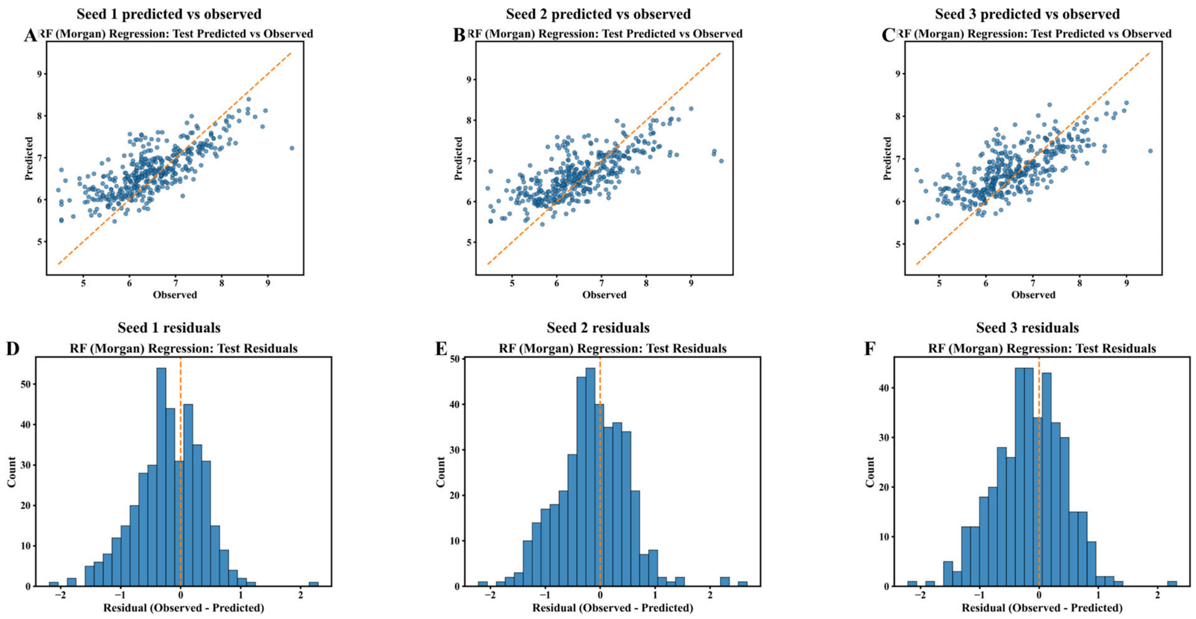

Figure S2. Per-seed random forest regression diagnostics for ACC2 pIC50 prediction. **A–C**, Predicted versus observed pIC50 values for the random forest Morgan-fingerprint regression model across scaffold-split seeds 1–3. **D–F**, Corresponding test-set residual distributions for each seed. Dashed lines in the predicted-versus-observed plots indicate the ideal prediction line, and dashed lines in the residual plots indicate zero residual.

Histogram gradient boosting regression diagnostics

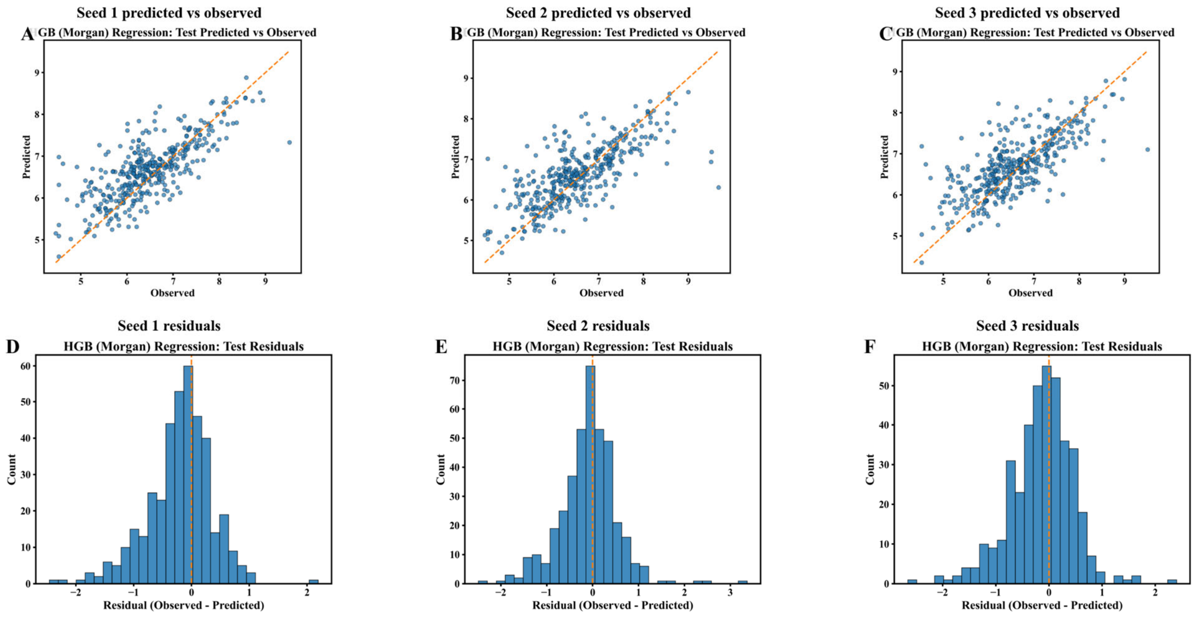

Figure S3. Per-seed histogram gradient boosting regression diagnostics for ACC2 pIC50 prediction. **A–C**, Predicted versus observed pIC50 values for the histogram gradient boosting Morgan-fingerprint regression model across

scaffold-split seeds 1–3. **D–F**, Corresponding test-set residual distributions for each seed. Dashed lines in the predicted-versus-observed plots indicate the ideal prediction line, and dashed lines in the residual plots indicate zero residual.

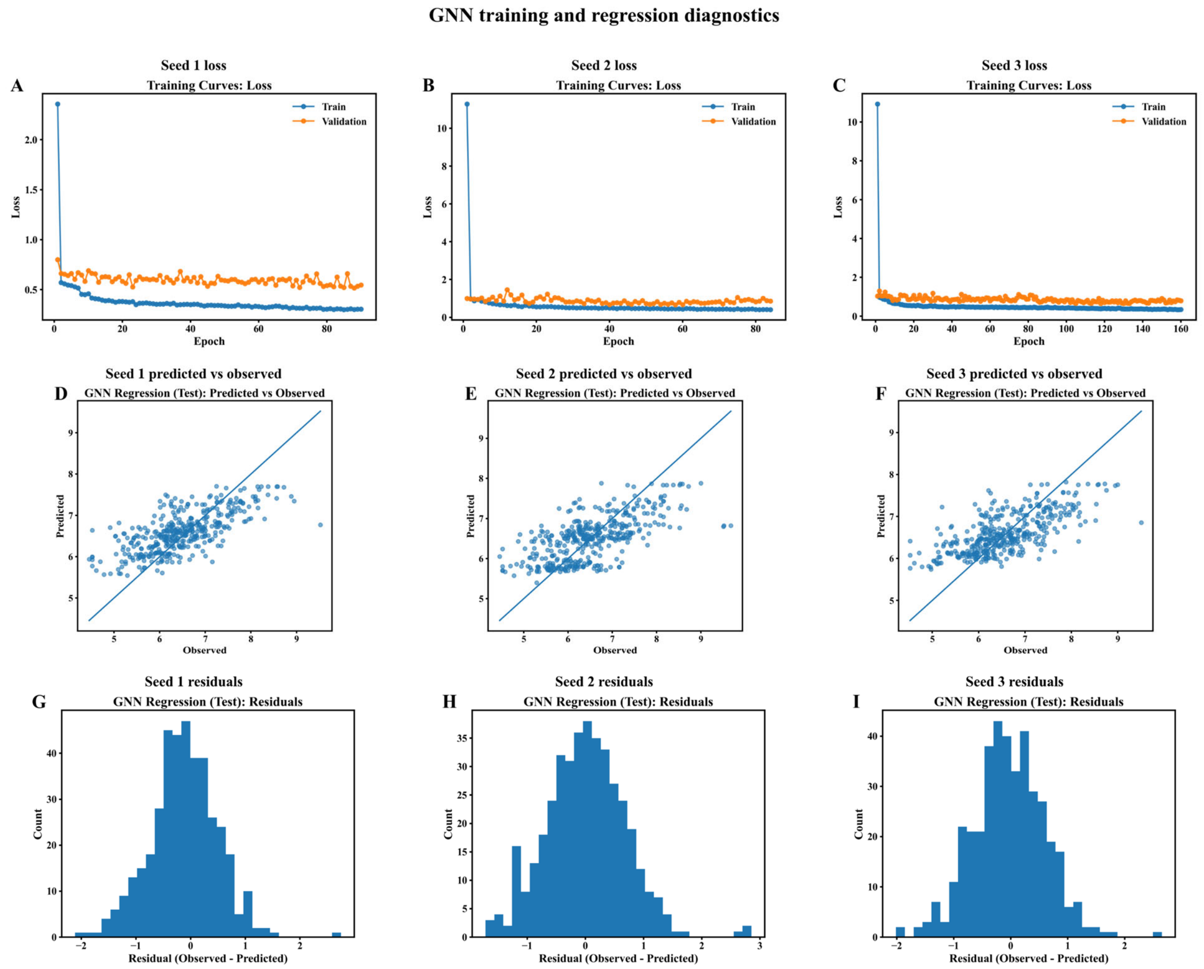

Figure S4. Training behaviour and per-seed regression diagnostics for the graph neural-network model. **A–C**, Training and validation loss curves for the graph neural-network regression model across scaffold-split seeds 1–3. **D–F**, Predicted versus observed pIC50 values on the test set for each seed. **G–I**, Corresponding test-set residual distributions. These diagnostics show model convergence and residual behaviour across independent scaffold splits.

GNN-fusion training and regression diagnostics

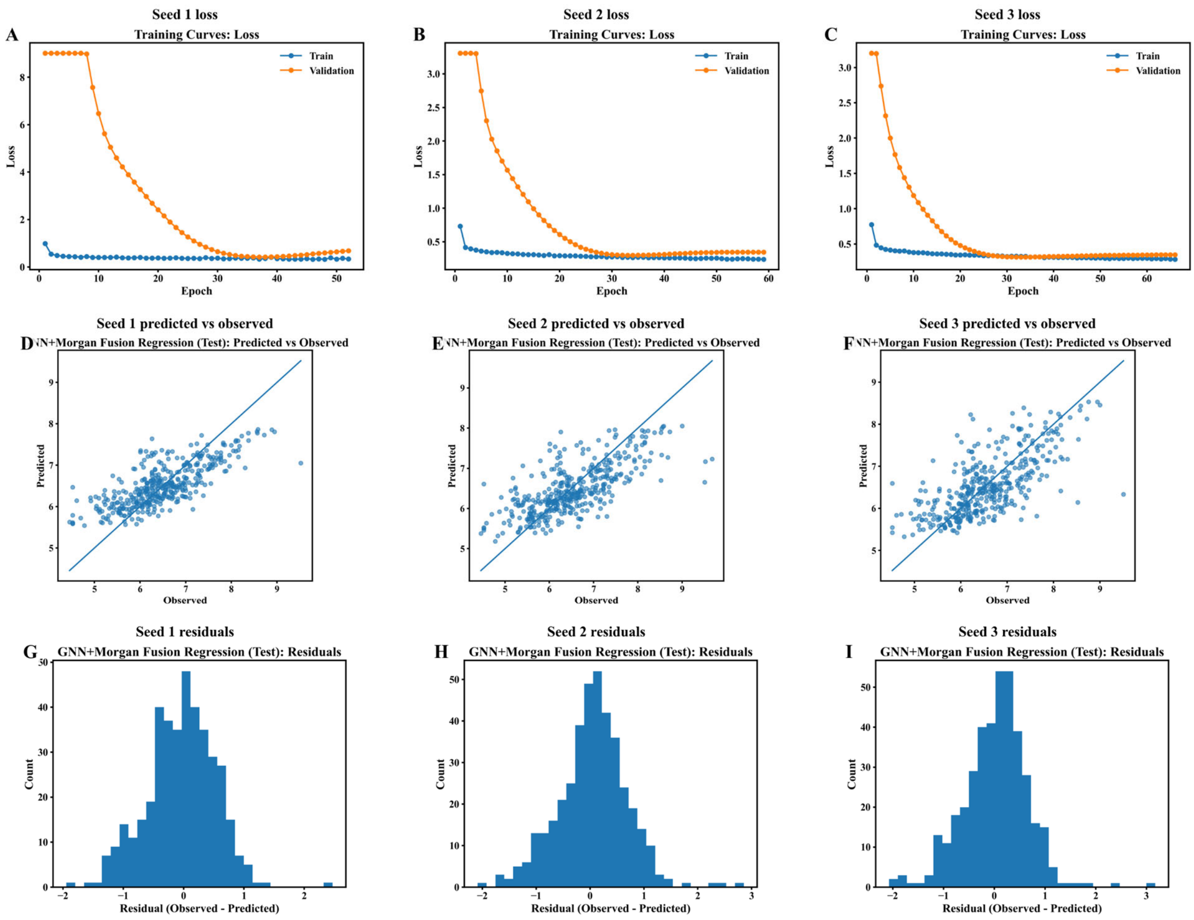

Figure S5. Training behaviour and per-seed regression diagnostics for the graph–Morgan fusion model. **A–C**, Training and validation loss curves for the graph–Morgan fusion regression model across scaffold-split seeds 1–3. **D–F**, Predicted versus observed pIC50 values on the test set for each seed. **G–I**, Corresponding test-set residual distributions. The plots summarize convergence, prediction agreement and residual structure for the late-fusion molecular representation.

Random forest classification diagnostics

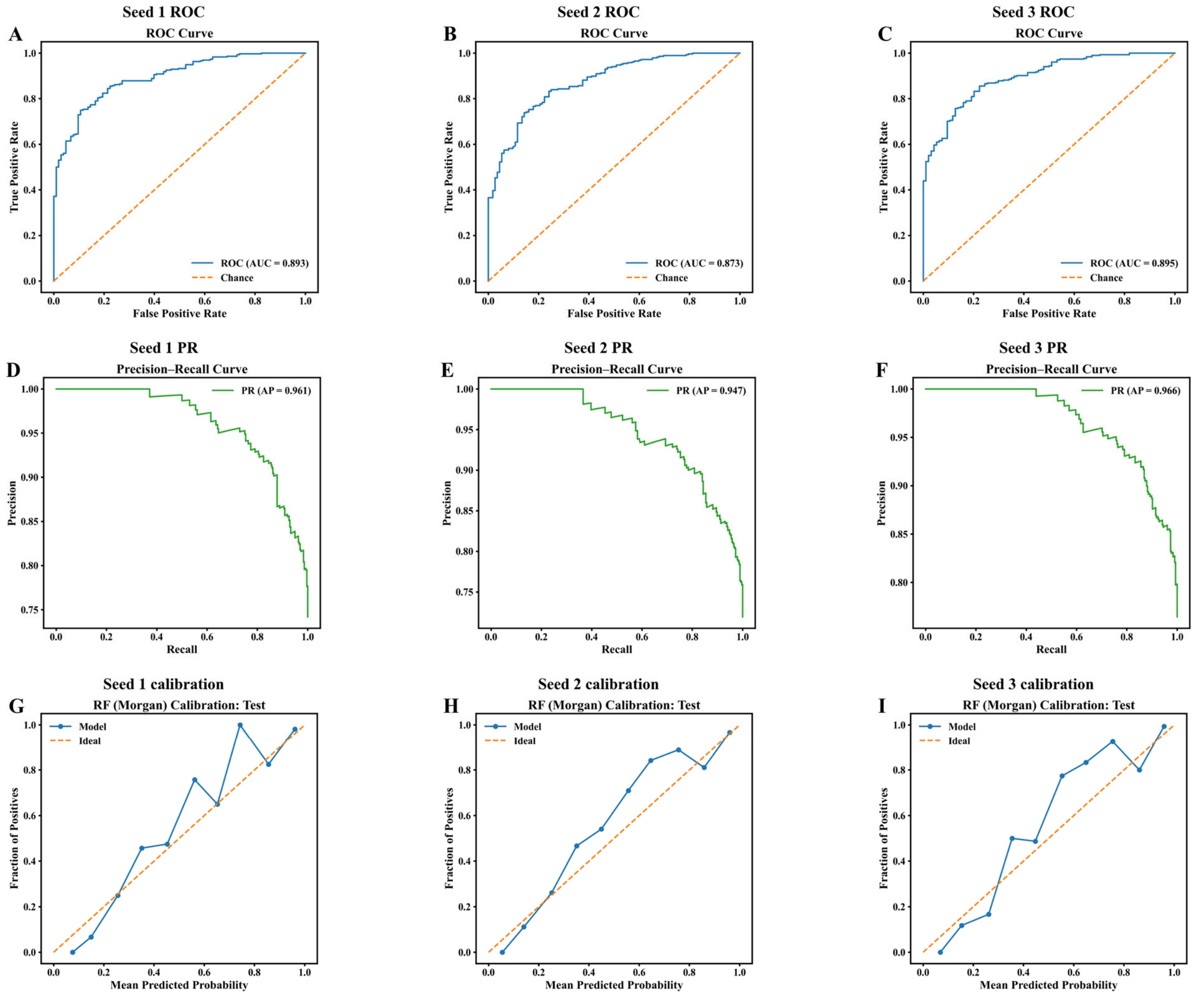

Figure S6. Per-seed random forest classification diagnostics for ACC2 activity prediction. **A–C**, Test receiver operating characteristic curves for the Morgan-fingerprint random forest classifier across scaffold-split seeds 1–3. **D–F**, Corresponding precision–recall curves. **G–I**, Test-set calibration plots comparing predicted probabilities with observed positive fractions for each seed.

Histogram gradient boosting classification diagnostics

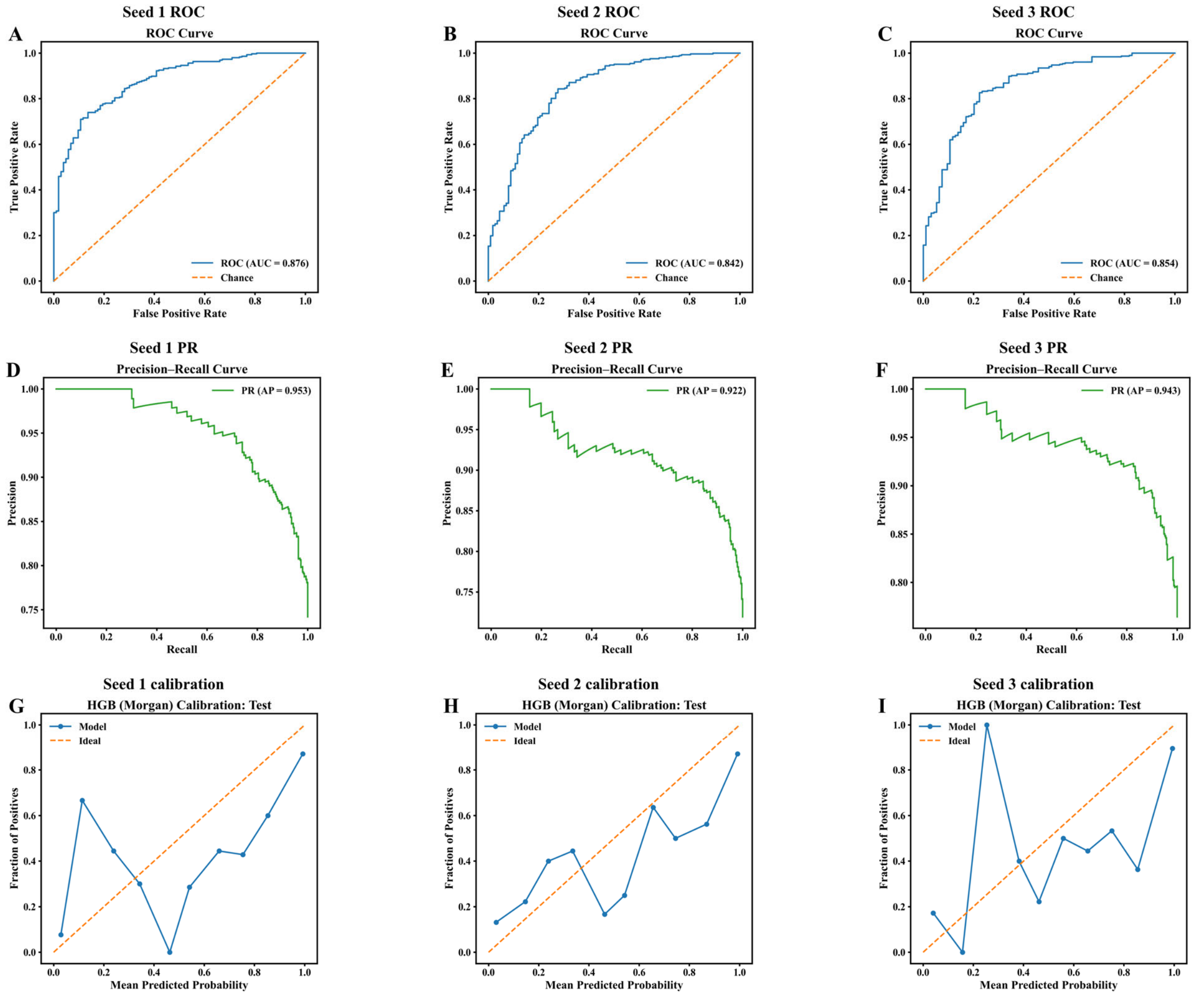

Figure S7. Per-seed histogram gradient boosting classification diagnostics for ACC2 activity prediction. **A–C**, Test receiver operating characteristic curves for the Morgan-fingerprint histogram gradient boosting classifier across scaffold-split seeds 1–3. **D–F**, Corresponding precision–recall curves. **G–I**, Test-set calibration plots for each seed.

GNN classification diagnostics

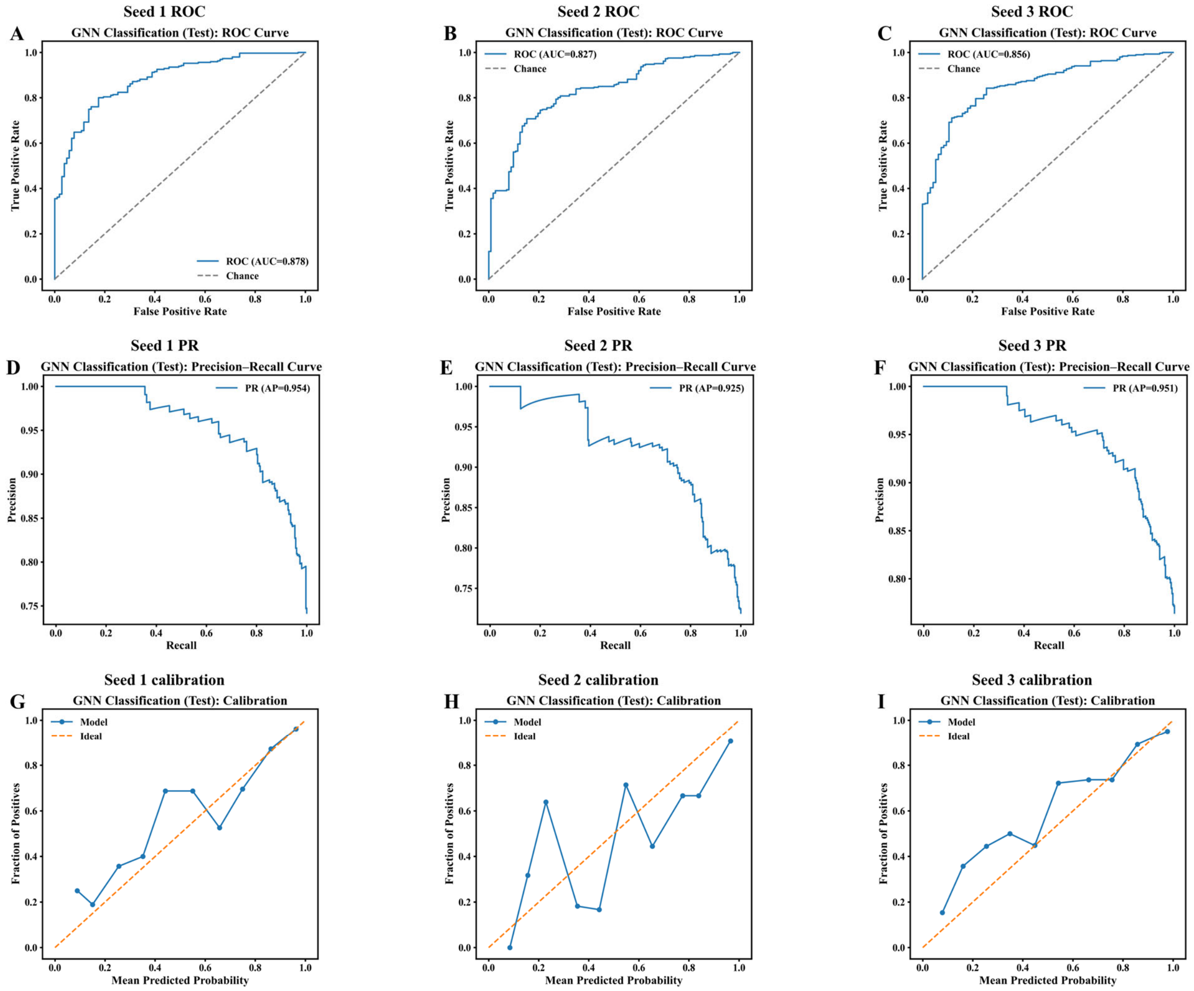

Figure S8. Per-seed graph neural-network classification diagnostics for ACC2 activity prediction. **A–C**, Test receiver operating characteristic curves for the graph neural-network classifier across scaffold-split seeds 1–3. **D–F**, Corresponding precision–recall curves. **G–I**, Test-set calibration plots for each seed.

GNN-fusion classification diagnostics

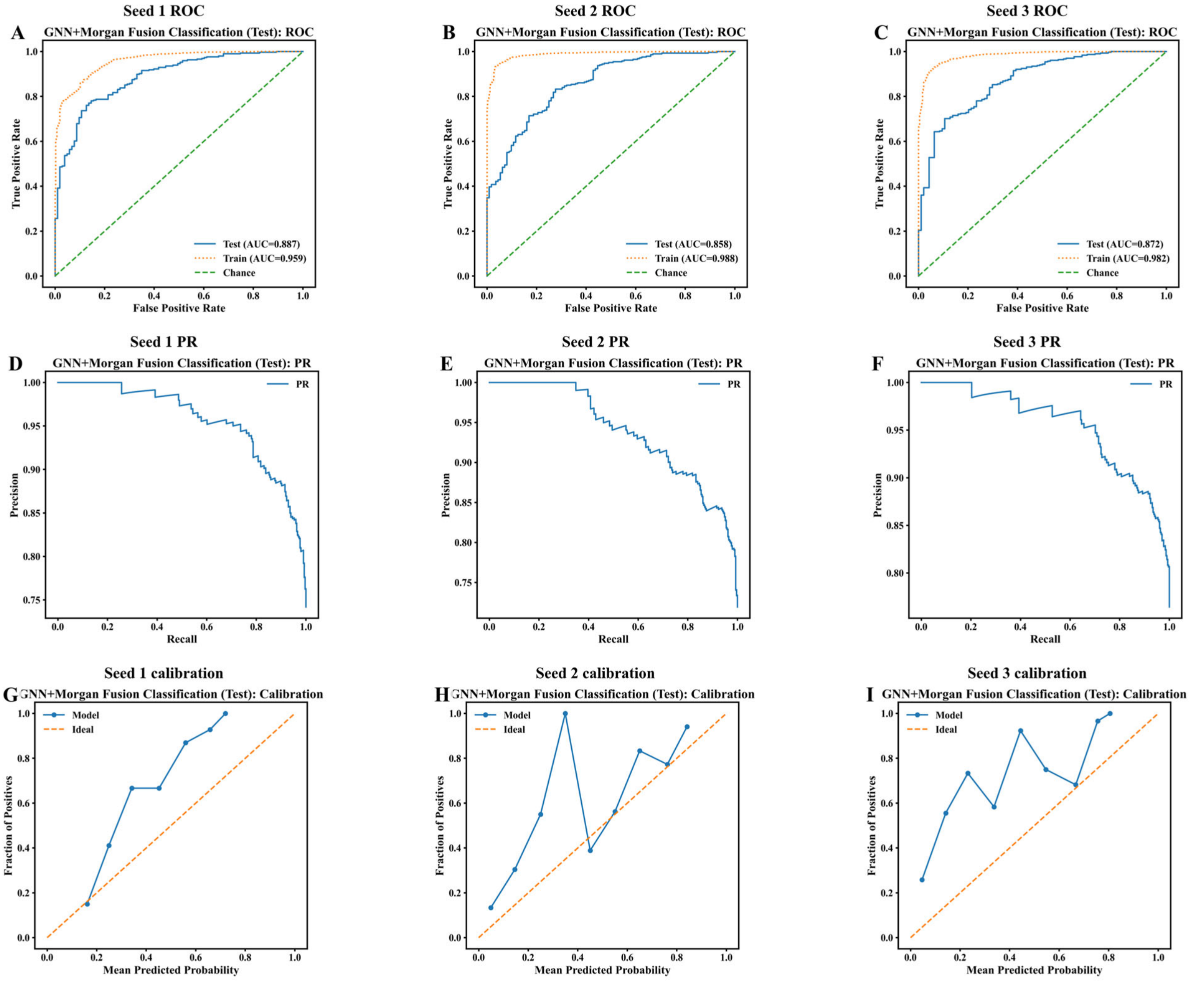

Figure S9. Per-seed graph–Morgan fusion classification diagnostics for ACC2 activity prediction. **A–C**, Train and test receiver operating characteristic curves for the graph–Morgan fusion classifier across scaffold-split seeds 1–3. **D–F**, Test-set precision–recall curves for each seed. **G–I**, Test-set calibration plots showing the relationship between predicted probabilities and observed positive fractions.

Platt calibration diagnostics for the GNN-fusion classifier

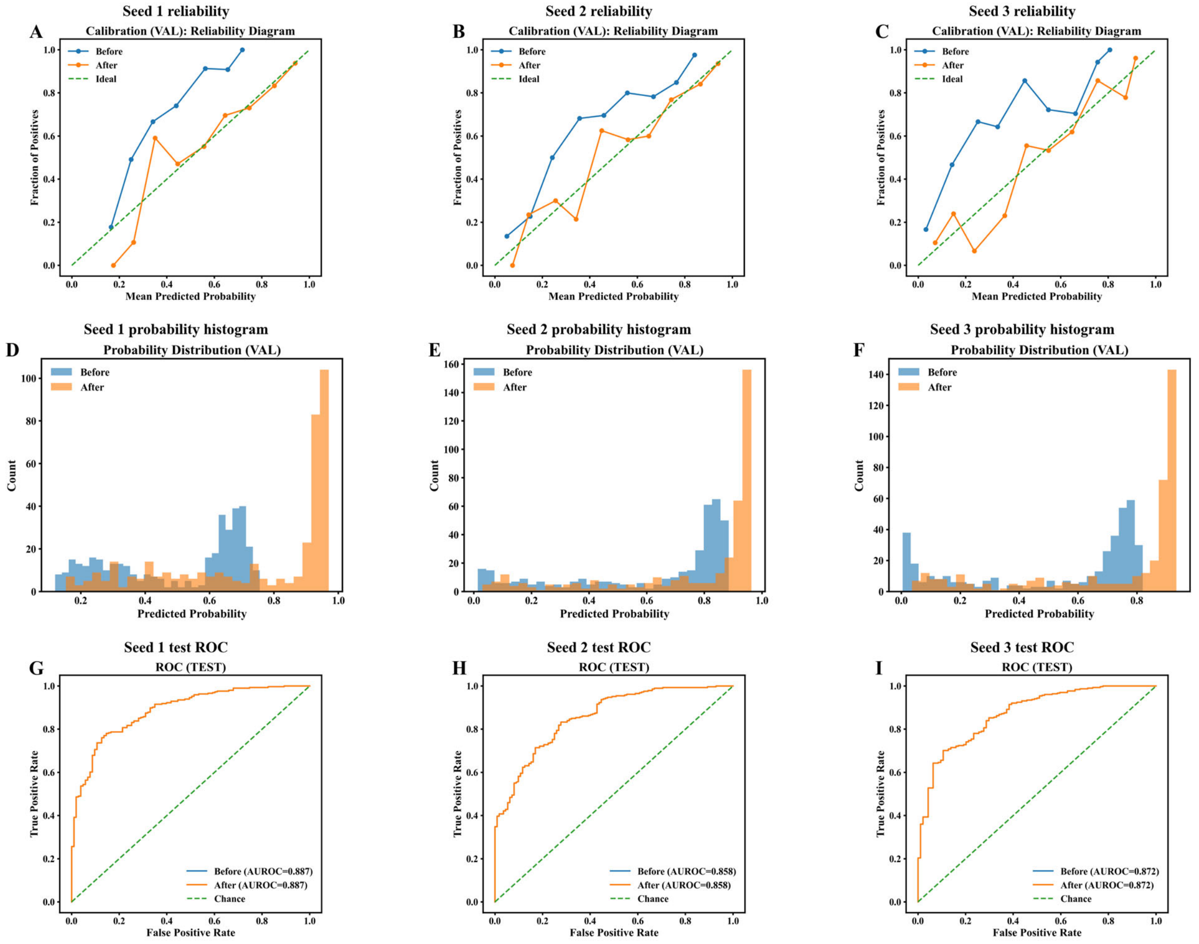

Figure S10. Validation-based Platt calibration of graph–Morgan fusion classification probabilities**. A–C**, Validation-set reliability diagrams before and after Platt scaling across scaffold-split seeds 1–3. **D–F**, Validation-set predicted-probability distributions before and after calibration. **G–I**, Test-set receiver operating characteristic curves before and after Platt scaling. Calibration changed the probability scale but preserved test-set discrimination.

Applicability-domain similarity and chemical-space diagnostics

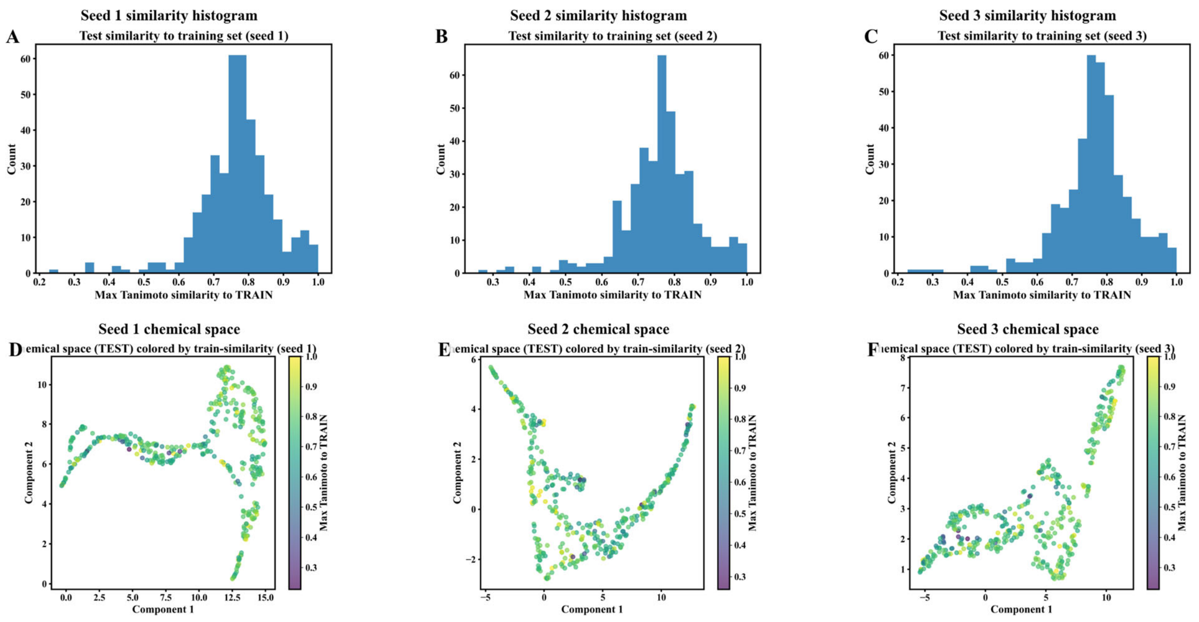

Figure S11. Test-set similarity to the ACC2 training set and chemical-space distribution across scaffold-split seeds**. A–C**, Distributions of maximum Morgan Tanimoto similarity between test compounds and training compounds for scaffold-split seeds 1–3. **D–F**, Two-dimensional test-set chemical-space projections coloured by maximum Tanimoto similarity to the training set for each seed.

Applicability-domain prediction-space diagnostics

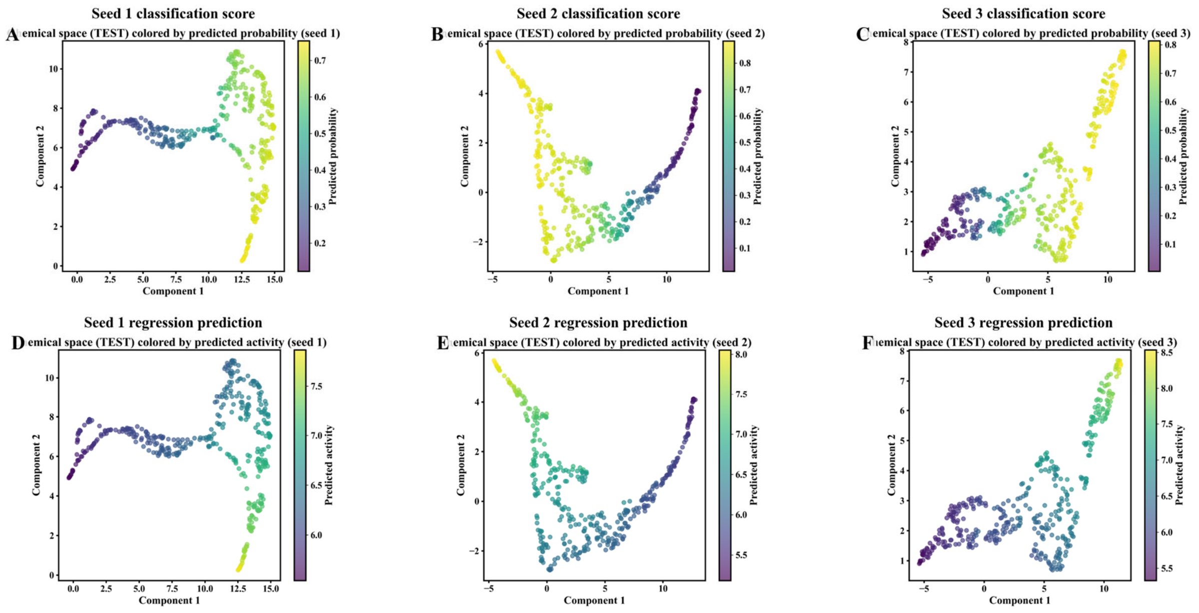

Figure S12. Chemical-space projections coloured by ACC2 model predictions. **A–C**, Two-dimensional test-set chemical-space projections coloured by predicted ACC2 activity probability for scaffold-split seeds 1–3. **D–F**,

Corresponding projections coloured by predicted pIC50 values. These plots show how classification and regression outputs vary across the scaffold-held-out chemical space.

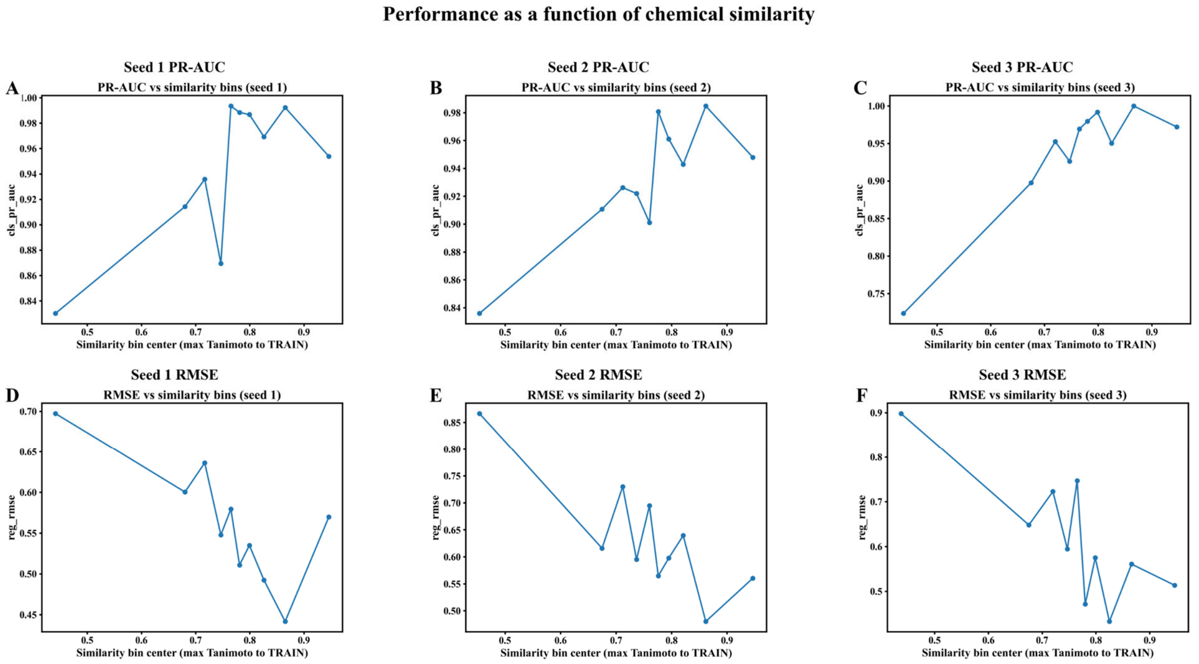

Figure S13. Classification and regression performance across nearest-neighbour similarity bins**. A–C**, Precision–recall area under the curve across maximum Tanimoto similarity bins for scaffold-split seeds 1–3. **D–F**, Root-mean-square error across the same similarity bins. Higher similarity to training compounds was generally associated with stronger classification performance and lower regression error.

SMILES: C[C@H](NC(=O)C1CC1)c1ccc(I)cc1

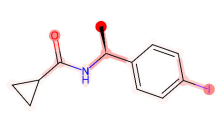

SMILES: CC(=O)N[C@@H](C)c1ccc(C2CN(c3nnc(-c4ccccc4…

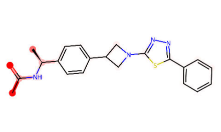

SMILES: COc1ccc(-c2ccccc2)cc1NS(=O)(=O)c1cc(Cl)cc(…

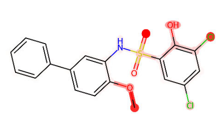

SMILES: CC(=O)NC(C)CCc1ccc(OC2=NCC(OC(C)C)N=C2)cc1

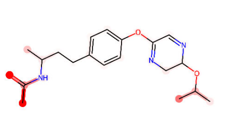

SMILES: CC(=O)N[C@@H](C)COc1ccc(Oc2ccc3c(c2Cl)CCN(…

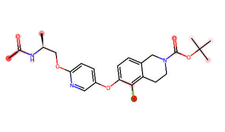

SMILES: CCN(CC)C(=O)c1cccc(N2CCN(C(=O)c3c4ccccc4cc…

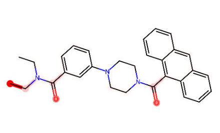

SMILES: CC(=O)NCCCC1OCC(c2cccc3cc(OC4CCCC4)ccc23)C…

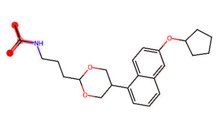

SMILES: CCN1Cc2ccc(N3CCN(C(=O)c4c5ccccc5cc5ccccc45…

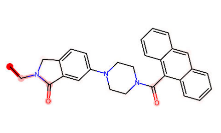

SMILES: CC(=O)NC(C)c1ccc(-c2ccc(Oc3ccc(-c4c(O)ccc5…

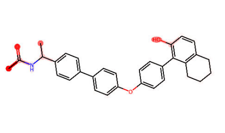

SMILES: CCNC(=O)Nc1sc2cc(C)ccc2c1C(=O)N1CCN(C2CCN(…

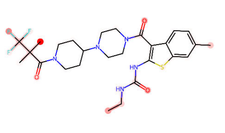

SMILES: O=C(c1cc(-c2ccccc2)nc2ccccc12)N1CCN(Cc2ccc…

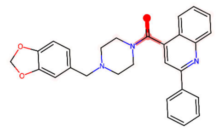

SMILES: CC(=O)NCCCC1OCC(Cc2cccc(OCCC3CC3)c2)CO1

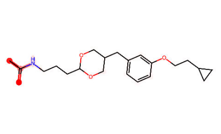

SMILES: CC(=O)N[C@@H](C)COc1ccc(Oc2ccc3c(c2Cl)CCN(…

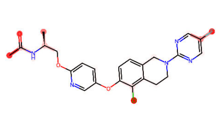

SMILES: COc1ncc(C#Cc2ccc(C(C)CNC(=O)c3cn[nH]c3)cc2…

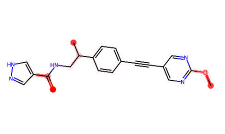

SMILES: CC(=O)NCCCC1OCC(Cc2cccc(OCCc3ccccc3)c2)CO1

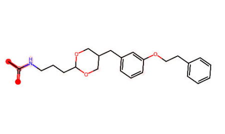

SMILES: CCOc1ccc(C#Cc2ccc(CN3CCCC3=O)cc2)cc1

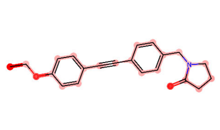

SMILES: O=C(c1c2ccccc2cc2ccccc12)N1CCC(N2CCC[C@@H]…

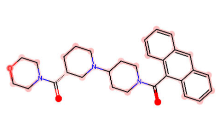

SMILES: CC(=O)NC(C)Cc1ccc(C2CN(c3ccc(-c4ccc(Cl)cc4…

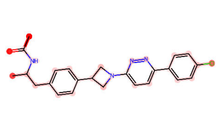

SMILES: CCOc1ccc(C#Cc2ccc(CC(C)N3CC(C)(C)C3=O)cc2)…

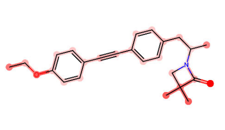

SMILES: CCN(CC)C(=O)c1cccc(N2CCN(C(=O)c3c4ccccc4cc…

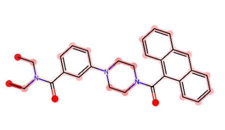

SMILES: COc1cc(C(=O)N2CCC3(CC2)CC(=O)c2cc(-c4cncc(…

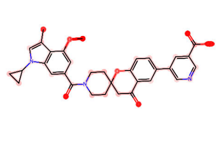

SMILES: CCNC(=O)Nc1sc2cc(C)ccc2c1C(=O)N1CCN(C2CCN(…

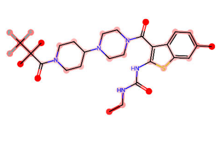

SMILES: Cn1cc(-c2cc3c(cn2)OC2(CCN(C(=O)c4ccc5[nH]c…

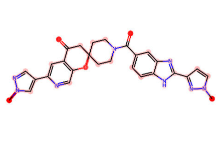

SMILES: CCOC(=O)c1sc2c(c1C)c(=O)n(CCO)[illegible]=O)n2CCc1c…

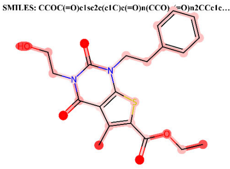

SMILES: CC(C)(C)OC(=O)NC[C@H]1CC[C@H](CNS(=O)(=O)c…

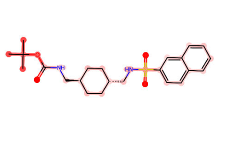

SMILES: O=C(c1cc(-c2ccccc2)nc2ccccc12)N1CCN(Cc2ccc…

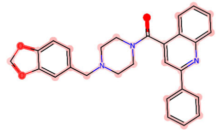

SMILES: CC(=O)NCCCC1OCC(Cc2cccc(OCCC3CC3)c2)CO1

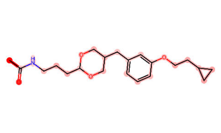

SMILES: CC(=O)N[C@@H](C)COc1ccc(Oc2ccc3c(c2Cl)CCN(…

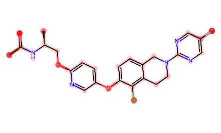

SMILES: C=C(CC(C)Cc1ccc(C#Cc2ccc(CCC)cc2)cc1)C1CC(…

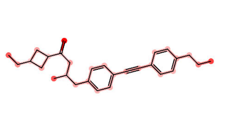

SMILES: CC(C)Oc1ccc(C2=NC(CSc3nc4ncccc4o3)CO2)cc1

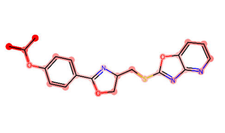

SMILES: Cc1nc2cc(C(=O)N3CCC4(CC3)CC(=O)c3c(c(C(=O)…

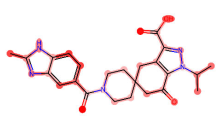

SMILES: O=C(c1c2ccccc2cc2ccccc12)N1CCC(N2CCC[C@@H]…

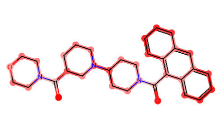

SMILES: CC(=O)N[C@@H](C)c1ccc(C2CN(c3nnc(-c4ccccc4…

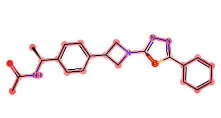

SMILES: CC(=O)N1CCC(C(=O)N2CCC(N3CCN(C(=O)c4cc(-c5…

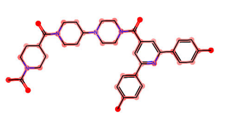

SMILES: COc1ccc(-c2ccccc2)cc1NS(=O)(=O)c1cc(Cl)cc(…

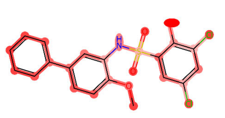

SMILES: CC(=O)NC(C)Cc1ccc(C2CN(c3ccc(-c4ccc(Cl)cc4…

SMILES: CC(=O)NCCCC1OCC(c2cccc3cc(OC4CCCC4)ccc23)C…

SMILES: COc1cc(C(=O)N2CCC3(CC2)CC(=O)c2cc(-c4cncc(…

SMILES: CCN1Cc2ccc(N3CCN(C(=O)c4c5ccccc5cc5ccccc45…

SMILES: CCOC(=O)c1sc2c(c1C)c(=O)n(CCO)[illegible]=O)n2CCc1c…

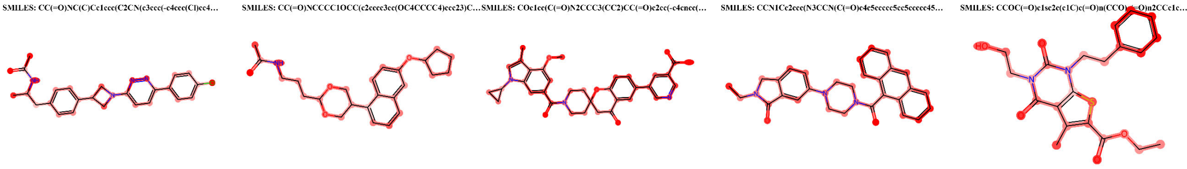

SMILES: CC(=O)NCCCC1OCC(Cc2cccc(OCC3CCC3)c2)CO1

SMILES: CC(=O)N[C@@H](C)COc1ccc(Oc2ccc3c(c2Cl)CCN(…

SMILES: CC(=O)NCCCC1OCC(Cc2cccc(OCCc3ccccc3)c2)CO1

SMILES: CC(C)Oc1ccc(C2=NC(CSc3nc4ncccc4o3)CO2)cc1

SMILES: CC(=O)N[C@@H](C)CO[C@H]1C[C@H](c2nc3cnc(OC…

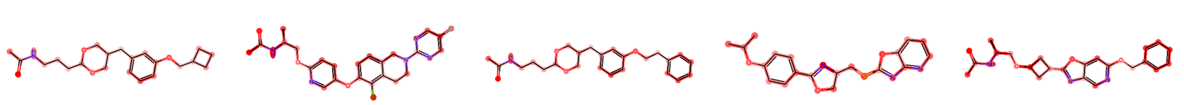

Figure S14. Representative atom-level attribution maps from the graph–Morgan fusion model. Atom-level attribution maps are shown for representative test compounds selected from the interpretability analysis. Highlighted atoms indicate molecular regions contributing to the model output for the displayed prediction task. These maps describe model-attribution patterns and do not establish experimentally validated ACC2 binding interactions.

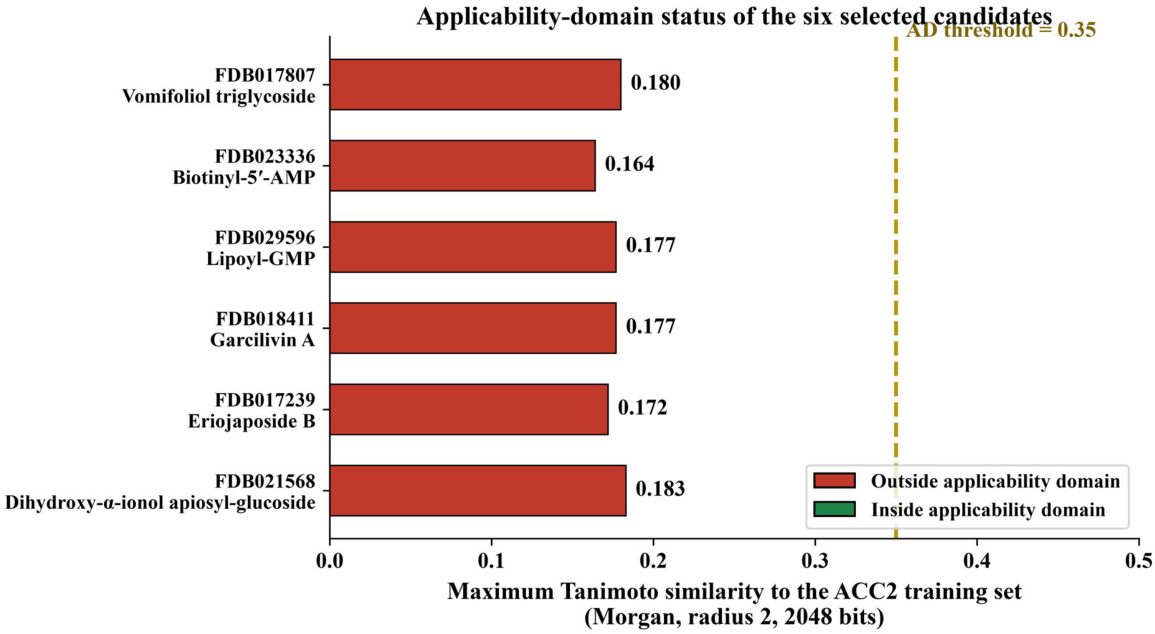

Figure 15. Applicability-domain status of the six selected FooDB compounds. Bars show the maximum Morgan–Tanimoto similarity (radius 2, 2048 bits) of each compound to the ACC2 training set; the dashed line marks the applicability-domain (AD) threshold of 0.35. All six compounds fall below the threshold and therefore lie outside the model's reliable applicability domain, indicating that their predicted ACC2 activities are extrapolations beyond the training chemical space.

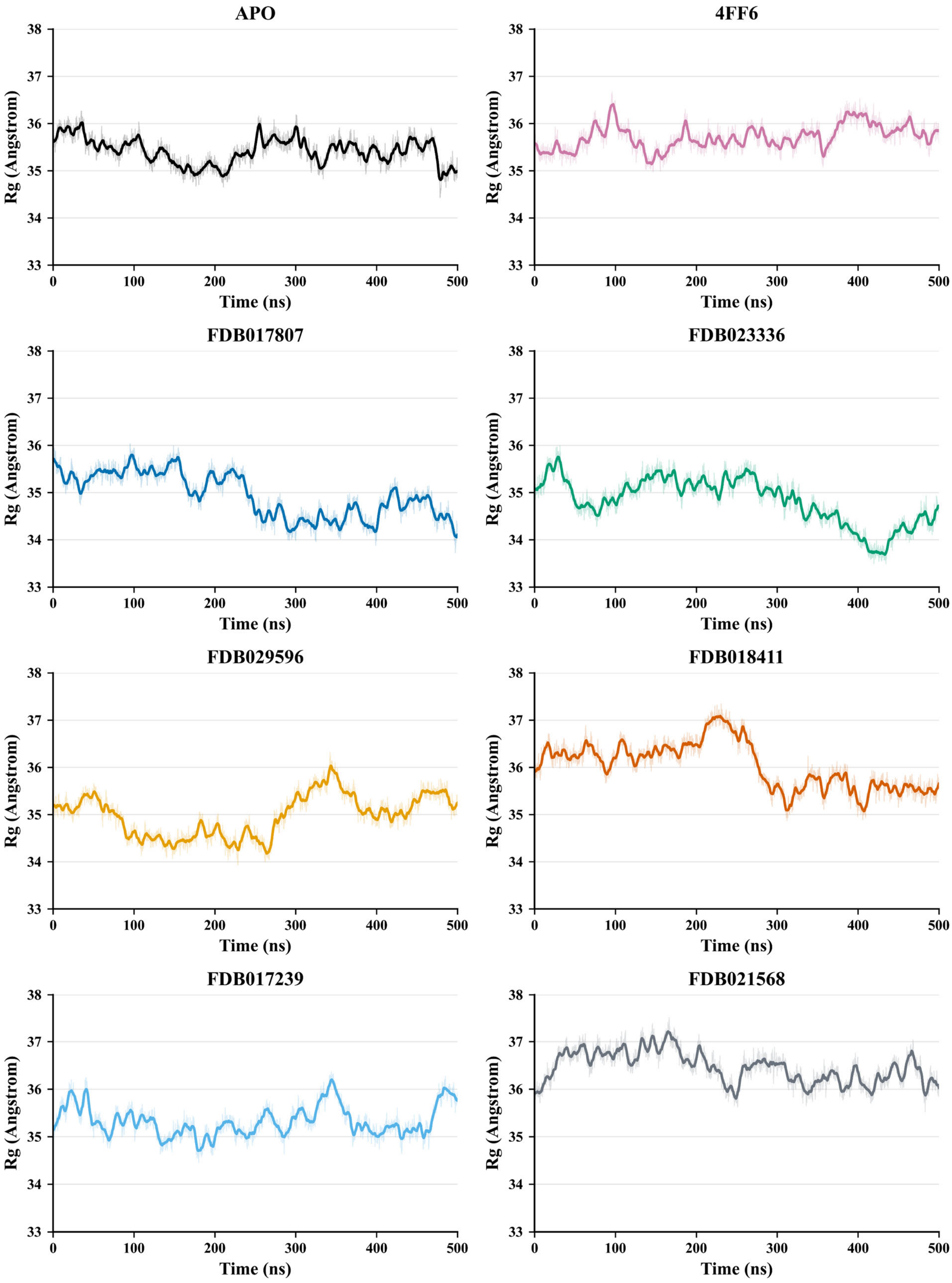
APO
4FF6
FDB017807
FDB023336
FDB029596
FDB018411
FDB017239
FDB021568
Rg (Angstrom)
Time (ns)
38
37
36
35
34
33
0
100
200
300
400
500

Figure S16. Radius of gyration (Rg) trajectories of apo ACC2, the reference-ligand complex (3FF6), and ACC2 bound to six selected FooDB compounds over 500 ns simulations.

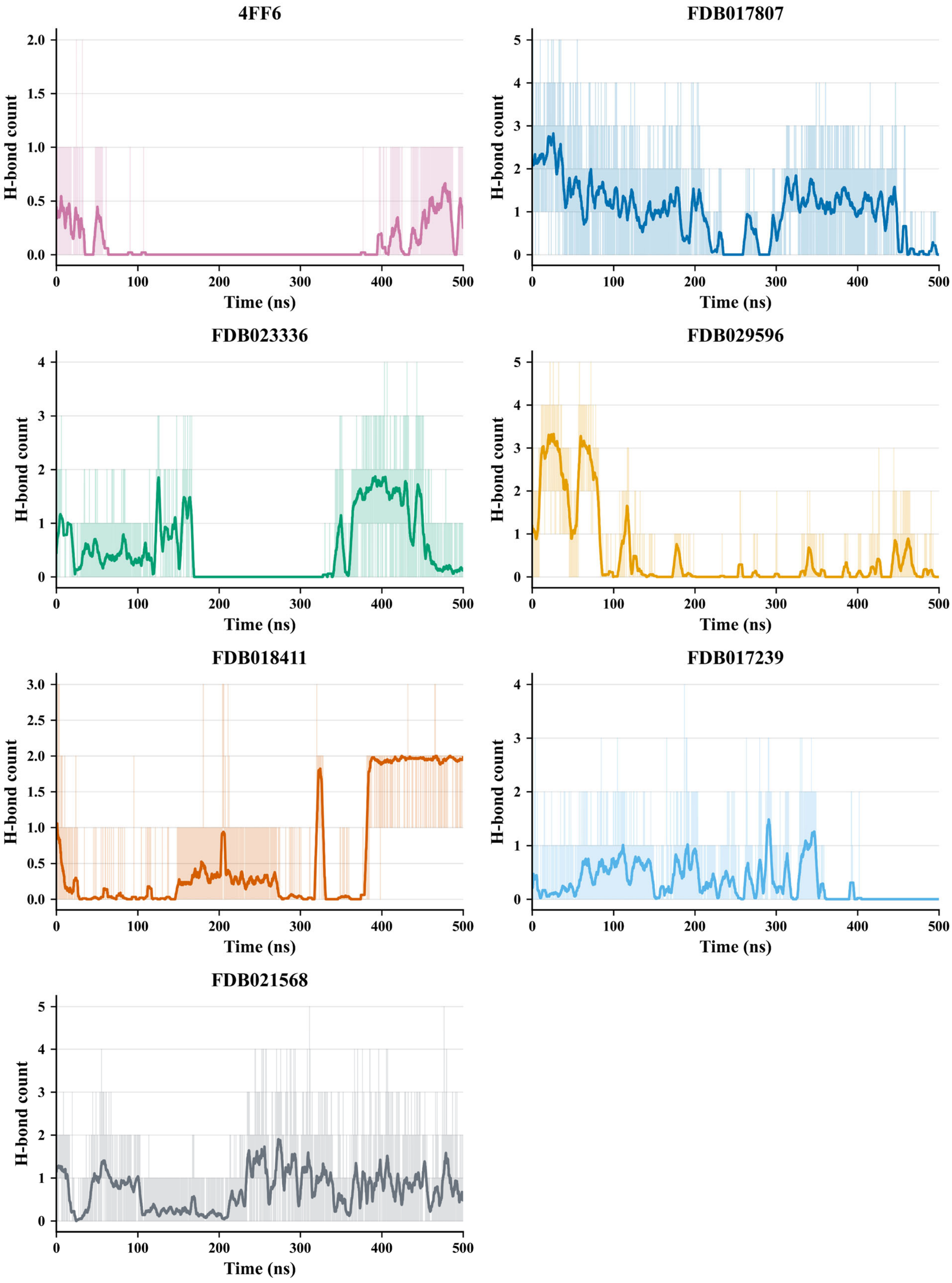
4FF6
FDB017807
FDB023336
FDB029596
FDB018411
FDB017239
FDB021568
H-bond count
Time (ns)
0
100
200
300
400
500

Figure S17. Number of hydrogen bonds formed between ACC2 and the reference ligand (3FF6) and six selected FooDB compounds over 500 ns molecular dynamics simulations.

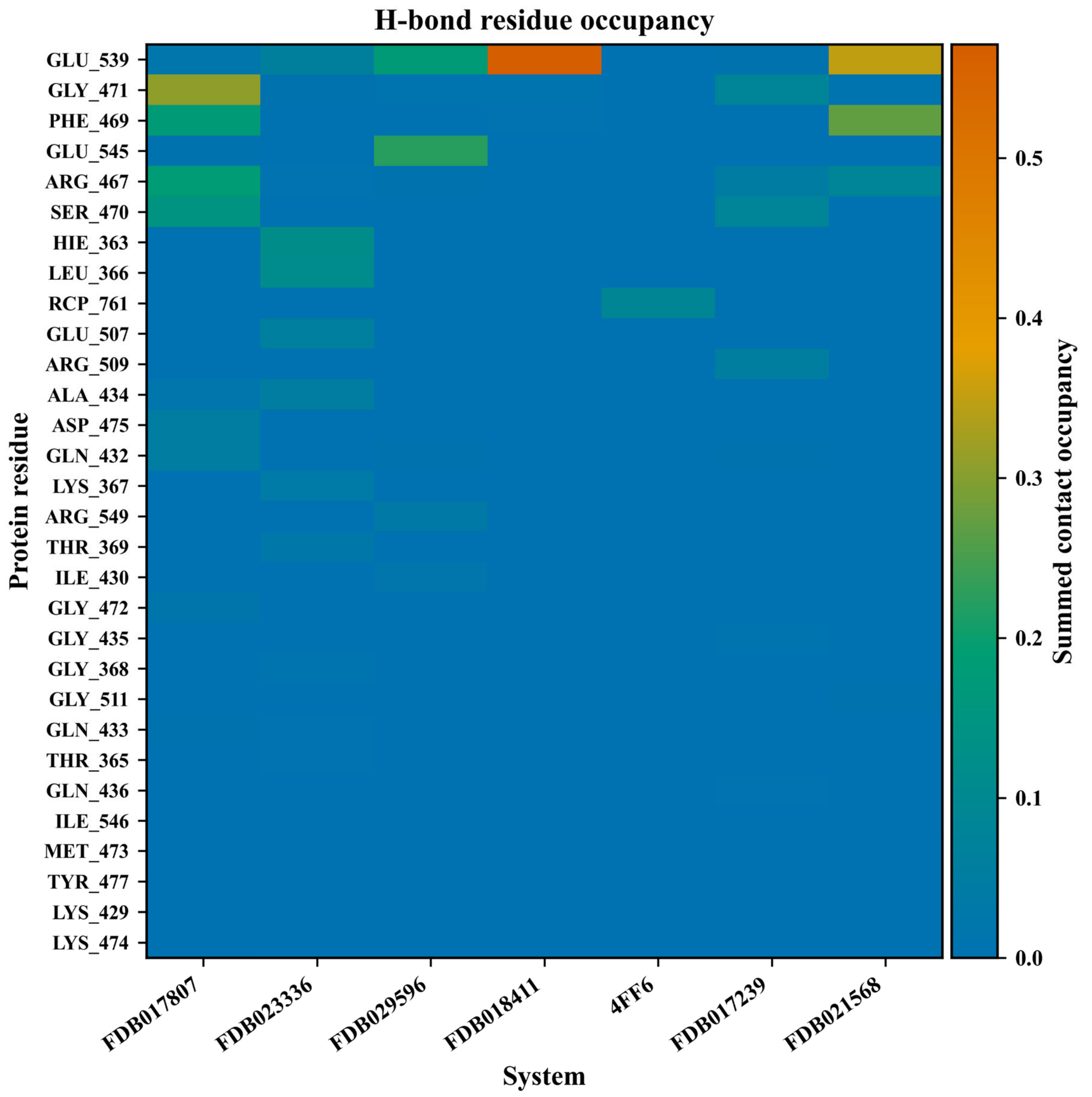

Figure S18. Heatmap showing hydrogen bond residue occupancy for the reference-ligand complex and six FooDB-bound ACC2 systems during molecular dynamics simulations. Rows represent ACC2 residues involved in ligand hydrogen bonding, and columns represent simulated systems. The colour scale indicates summed contact occupancy, with higher values corresponding to more persistent hydrogen bond interactions during the trajectory.
